# The multiple *de novo* copy number variant (M*dn*CNV) phenomenon presents with peri-zygotic DNA mutational signatures and multilocus pathogenic variation

**DOI:** 10.1186/s13073-022-01123-w

**Published:** 2022-10-27

**Authors:** Haowei Du, Angad Jolly, Christopher M. Grochowski, Bo Yuan, Moez Dawood, Shalini N. Jhangiani, He Li, Donna Muzny, Jawid M. Fatih, Zeynep Coban-Akdemir, Mary Esther Carlin, Angela E. Scheuerle, Karin Witzl, Jennifer E. Posey, Matthew Pendleton, Eoghan Harrington, Sissel Juul, P. J. Hastings, Weimin Bi, Richard A. Gibbs, Fritz J. Sedlazeck, James R. Lupski, Claudia M. B. Carvalho, Pengfei Liu

**Affiliations:** 1 Department of Molecular and Human Genetics, Baylor College of Medicine, One Baylor Plaza, Houston, TX 77030 USA; 2 Medical Scientist Training Program, Baylor College of Medicine, Houston, TX 77030 USA; 3 Baylor Genetics Laboratory, Houston, TX 77021 USA; 4 Seattle Children’s Hospital, Seattle, WA 98105 USA; 5 Human Genome Sequencing Center, Baylor College of Medicine, Houston, TX 77030 USA; 6 Human Genetics Center, Department of Epidemiology, Human Genetics, and Environmental Sciences, School of Public Health, The University of Texas Health Science Center at Houston, Houston, TX 77030 USA; 7 Division of Genetics and Metabolism, Department of Pediatrics, University of Texas Southwestern Medical Center, Dallas, TX 75390 USA; 8 Division of Genetics Diagnostics, Department of Pathology, University of Texas Southwestern Medical Center, Dallas, TX 75390 USA; 9 McDermott Center for Human Growth and Development, University of Texas Southwestern Medical Center, Dallas, TX 75390 USA; 10 Clinical Institute of Medical Genetics, University Medical Centre Ljubljana, 1000 Ljubljana, Slovenia; 11 Medical Faculty, University of Ljubljana, 1000 Ljubljana, Slovenia; 12 Oxford Nanopore Technologies Inc, New York, NY 10013 USA; 13 Dan L. Duncan Comprehensive Cancer Center, BCM, Houston, TX 77030 USA; 14 Department of Pediatrics, Baylor College of Medicine, Houston, TX 77030 USA; 15 Texas Children’s Hospital, Houston, TX 77030 USA; 16 Pacific Northwest Research Institute, 720 Broadway, Seattle, WA 98122 USA

**Keywords:** Long-read sequencing, Genomic data integration, Genomic data visualization, MMBIR, Genomic instability, Tandem duplication, *De novo *CNV, *De novo* SNV, Human Phenotype Ontology, Structural variation

## Abstract

**Background:**

The multiple *de novo* copy number variant (M*dn*CNV) phenotype is described by having four or more constitutional *de novo* CNVs (*dn*CNVs) arising independently throughout the human genome within one generation. It is a rare peri-zygotic mutational event, previously reported to be seen once in every 12,000 individuals referred for genome-wide chromosomal microarray analysis due to congenital abnormalities. These rare families provide a unique opportunity to understand the genetic factors of peri-zygotic genome instability and the impact of *dn*CNV on human diseases.

**Methods:**

Chromosomal microarray analysis (CMA), array-based comparative genomic hybridization, short- and long-read genome sequencing (GS) were performed on the newly identified M*dn*CNV family to identify *de novo* mutations including *dn*CNVs, *de novo* single-nucleotide variants (*dn*SNVs), and indels. Short-read GS was performed on four previously published M*dn*CNV families for *dn*SNV analysis. Trio-based rare variant analysis was performed on the newly identified individual and four previously published M*dn*CNV families to identify potential genetic etiologies contributing to the peri-zygotic genomic instability. Lin semantic similarity scores informed quantitative human phenotype ontology analysis on three M*dn*CNV families to identify gene(s) driving or contributing to the clinical phenotype.

**Results:**

In the newly identified M*dn*CNV case, we revealed eight *de novo* tandem duplications, each ~ 1 Mb, with microhomology at 6/8 breakpoint junctions. Enrichment of *de novo* single-nucleotide variants (SNV; 6/79) and *de novo* indels (1/12) was found within 4 Mb of the *dn*CNV genomic regions. An elevated post-zygotic SNV mutation rate was observed in M*dn*CNV families. Maternal rare variant analyses identified three genes in distinct families that may contribute to the M*dn*CNV phenomenon. Phenotype analysis suggests that gene(s) within *dn*CNV regions contribute to the observed proband phenotype in 3/3 cases. CNVs in two cases, a contiguous gene duplication encompassing *PMP22* and *RAI1* and another duplication affecting *NSD1* and *SMARCC2*, contribute to the clinically observed phenotypic manifestations.

**Conclusions:**

Characteristic features of *dn*CNVs reported here are consistent with a microhomology-mediated break-induced replication (MMBIR)-driven mechanism during the peri-zygotic period. Maternal genetic variants in DNA repair genes potentially contribute to peri-zygotic genomic instability. Variable phenotypic features were observed across a cohort of three M*dn*CNV probands, and computational quantitative phenotyping revealed that two out of three had evidence for the contribution of more than one genetic locus to the proband’s phenotype supporting the hypothesis of *de novo* multilocus pathogenic variation (MPV) in those families.

**Supplementary Information:**

The online version contains supplementary material available at 10.1186/s13073-022-01123-w.

## Background

*De novo* copy number variants (*dn*CNVs) that occur during gametogenesis or early post-zygotic development are present in all or most cells of a multicellular organism. Genome-wide surveys of large populations estimate the *de novo* mutation rate for structural variants to be 0.16–0.29 events per generation in humans [1, 2]. The rate for *dn*CNVs with a length over 100 kb is lower, around 0.012 per haploid genome [3]. A unique mutational phenomenon described by Liu et al. highlighted individuals with variable congenital abnormalities and multiple (*n* ≥ 4) *dn*CNVs (M*dn*CNV) throughout their genome [4]. The M*dn*CNV event, or phenomenon, can encompass several genes at each CNV locus [4].

M*dn*CNV is a rare mutational phenomenon, identified in only 5/60,000 individuals referred for genome-wide chromosomal microarray analysis [4]. M*dn*CNV has likely been under-appreciated due to the limited genomic resolution in clinical testing. The prominent features of *dn*CNVs shared between M*dn*CNV cases include (1) a predominance of copy number gains across multiple chromosomes, (2) tandem duplications forming the majority of copy number gains, (3) the presence of sequence microhomology or microhomeology at breakpoint junctions, and (4) other mutational signatures of SV mutagenesis such as the DUP-TRP-DUP pattern of Complex Genomic Rearrangement (CGR) [4].

The variable congenital abnormalities observed in individuals exhibiting the M*dn*CNV phenomenon are thought to be caused by the copy number change of different critical driver gene(s) in each proband. The Human Phenotype Ontology (HPO) represents a structured language database of human phenotype terms that allows for numerical coding of clinical phenotypes as HPO terms; such phenotypes may be observed in a proband or described in association with a gene or rare disease trait clinical synopsis in OMIM [5]. This numerical coding enables quantitative, computational analyses of a patient’s phenotypic features and comparison with phenotype associations within the literature to inform genomic variant prioritization.

Here we report a new family with multiple *dn*CNVs and leverage multiple genomic and phenotypic methodologies combined with visualization tools to extend our understanding of the M*dn*CNV mutational phenomenon. Rare variant and mutational signature analyses on the newly described and four previously characterized M*dn*CNV families suggest a maternal genetic variant contributing to peri-zygotic genome instability. Gene content of the affected genomic regions was analyzed using HPO to identify potential driver gene(s) and explore the hypothesis that the observed trait manifestation may be driven by multilocus pathogenic variation (MPV).

## Methods

### Subjects

The newly identified M*dn*CNV family (HOU3579) was initially ascertained through clinical chromosomal microarray analysis (CMA) performed at Baylor Genetics. Written consents were obtained for the proband (BAB9637), unaffected siblings (BAB9640, BAB9641, and BAB9642), and parents (BAB9638 and BAB9639) to perform further genomic studies. Oligo array-based comparative genomic hybridization (aCGH) was performed on all family members (“Methods” section). Illumina short-read (SR) whole-genome sequencing (WGS) and long-read (LR) WGS with Oxford Nanopore Technologies (ONT) were performed on the proband and parents’ blood leukocyte-derived DNA (Additional file 1: Supplementary methods). PacBio LR genomic sequencing was performed on the proband alone (“Methods”). Illumina short-read WGS was performed on nine anonymized families under a separate IRB protocol with a waiver of consent, including four  previously reported M*dn*CNV families (BAB3097, BAB3596, mCNV3/BAB9484, and mCNV7) [4] and five additional families without a M*dn*CNV phenotype as controls (Additional file 1: Supplementary methods).

### Array comparative genomic hybridization

The family’s DNA samples were initially analyzed by a clinical chromosomal microarray analysis (CMA) designed and performed by Baylor Genetics (BG) [6, 7]. Subsequently, high-resolution aCGH, using a 1 million probe whole-genome oligonucleotide microarray (Agilent microarray design ID:085903), was performed on all family members. All array-based experiments were implemented according to the Agilent aCGH protocol for probe labeling and hybridization with minor modifications [8].

### Illumina SR sequencing

Genome sequencing was performed on proband and parents with an average read depth of 35 × at the Baylor College of Medicine Human Genome Sequencing Center. Please refer to supplementary material for the details.

### Pacific Biosciences (PacBio) LR sequencing data

PacBio LR genomic sequencing was performed on the proband alone. The sequencing library was constructed with 5 µg genomic DNA using the SMRTbell Express Template Preparation Kit with an average insert size of 7.5 kb. The library was sequenced with five SMRTcells using the PacBio Sequel I instrument, which yielded 42 Gb of data.

### Nanopore trio LR sequencing and mapping

LR sequencing libraries were generated according to standard Oxford Nanopore Technologies (ONT) protocols. Detailed sequencing process and metrics are provided (Additional file 1: Supplementary methods and Table S1). Average coverage of 25 × was achieved for the trio.

### *De novo* single-nucleotide variant (SNV) and indel variant calling

Individual germline SNVs and indels were called using GATK (v.4.1.3) haplotypecaller and the HGSC xAtlas variant calling pipelines [9]. The “-GVCF” option was used for the GATK haplotypecaller, which outputs a gVCF file that includes reference or variant sequence information for all nucleotide positions. Using recalibrated posterior genotype probabilities to allow rigorous calling, *de novo* variants (GQ ≥ 20 for all trio members) were annotated. All possible *de novo* variants were further annotated using DNM (*de novo m*utation)-Finder (https://github.com/BCM-Lupskilab/DNM-Finder) [10], and manual inspection with Integrative Genomics Viewer (IGV) software was applied to confirm or reject the variant. It was performed with the following criteria per variant: (1) supported by at least 5 uniquely mapped reads; (2) supported by both forward and reverse strand reads; (3) variant did not derive from misalignment at indel variants; (4) not located at highly repetitive regions masked by RepeatMasker file for reference genome GRCh38 extracted from UCSC browser. In addition, potential *de novo* SNVs mapping within 20 bp of each other (clustered SNVs) was error-prone and thus removed from this study. *De novo* substitutions with variant read to total read ratio less than 0.35 or more than 0.65 (VR/TR < 0.35 or VR/TR > 0.65 and no read detected in any parental samples to exclude potential mosaicism in the parental genome) were classified as possible post-zygotic variant allele events.

### Mutational pattern analysis

The R/Bioconductor package MutationalPatterns [11] was used for mutational signatures analysis on *dn*SNVs. The “cos_sim_matrix” function within the MutationPatterns package was used to calculate the cosine similarity between known COSMIC (v3.2) signatures [12] with the base substitution profile of M*dn*CNV families (Additional file 2: Table S2). To avoid overfitting, an unbiased refitting procedure was used to select the optimal combination of signatures using “fit_to_signatures_strict” function with “best_subset” method option. In short, the refitting process starts with a subset of signatures and then removes the signature that has the least contribution. The removal happens iteratively until it gets to the optimal subset.

### Phasing SNVs and CNVs with combined read-based and pedigree-based method

The read-based phasing was performed using the publicly available WhatsHap tool [13]. In-house developed R scripts (https://github.com/BCM-Lupskilab/PhaseDenovo) were used to perform pedigree-based genetic phasing of physical haplotype blocks. The script assigns parental origin to the physical haplotype block if there are ≥ 20 informative SNPs present, and ≥ 90% of them are consistent with a single parental origin. The *dn*SNVs were assigned to the parental chromosome based on segregated haplotype blocks. The *dn*CNVs were phased using the method as previously described [14].

### Structural variant (SV) calling and analyses

Binary sequence alignment (BAM) files from SR and LR sequencing were used for SV calling. For Illumina SR, SVs were called and genotyped using Parliament2 [15]. Sniffles [16] was used for LR SV calling on the proband using the following parameters to maximize sensitivity: “ -s 8”. The SV calls from the proband were genotyped based on parental BAM files using Sniffles with the option “–Ivcf”. Subsequently, SVs called exclusively in the proband were filtered with SURVIVOR allowing a maximum distance of 1000 bp between pairwise breakpoint junction calls from each algorithm. For *dn*CNVs, the log2 ratio of read depth was visualized and manually examined (See “Visualization of genomics data for dnCNVs”) to minimize false-positive calls.

### Breakpoint junction amplification and sequencing analysis

Soft-clipped reads overlapping breakpoint junctions were extracted from LR sequence alignment files and remapped to the human genome (GRCh38) with the UCSC BLAT tool to single base-pair resolution. The amplified breakpoint junctions were confirmed by Sanger dideoxynucleotide sequencing. The presence of non-B DNA-forming sequence motifs, including Z-DNA, G-quadruplex, A-phased repeats, inverted repeats, mirror repeats, and direct repeats, were screened for within 50 bp to either side of the breakpoint junctions using the reference genome and the nBMST tool [17].

### *De novo* mutation rate estimation and statistical analysis for SNV clustering

The unphased genome-wide mutation rate per base-pair was estimated by taking the total number of DNMs divided by the size of the mappable diploid human reference genome (GRCh38). The phased genome-wide mutation rate was estimated by taking the total number of phased DNMs divided by the size of the mappable haploid human reference genome. The mappable human genome size (2.91 Tb/haploid genome) was computed using faCount from Kent’s tool (http://hgdownload.soe.ucsc.edu/admin/exe/linux.x86_64/). The number of phased DNMs to each haploid type was extrapolated based on the ratio of successfully phased DNMs. The interval defined was centered on the ~ 1 Mb *dn*CNV with an additional 4 Mb window size flanking either side, representing ~ 9 Mb intervals in total for an individual *dn*CNV. The dynamic window size ranged from 1 to 10 Mb. The observed count of DNMs in eight *dn*CNV regional intervals was calculated in reference to the size of the window range used. The corresponding Poisson probability was calculated by multiplying the genome-wide DNM density by the corresponding *dn*CNV interval length. The average count of DNMs at the same genomic intervals from 2976 genome sequenced trios [18] was calculated as the regional density control. The Poisson probability of observing the same or more DNMs was calculated using the ppois function from the R base package to determine the *p*-value.

### Visualization of genomics data for *dn*CNVs

The average read depth from SR sequencing was calculated using mosdepth v0.2.3 with the “– by 1000” option. We used the median read depth of each chromosome to normalize the read depth of that chromosome and calculated the log2 ratio for each 1000-bp window. The log2 ratio profiles were segmented using the Circular Binary Segmentation (CBS) algorithm [19] implemented in the DNAcopy Bioconductor package. The individual and segmented ratios were visualized together across genomic coordinates using KaryoploteR (v.1.16.0) [20] with log2 ratios of 0 representing normal copy number state, > 0.58 representing copy number gains, and <  − 1 representing copy number loss.

### Quantitative phenotyping analyses

To perform quantitative phenotype analysis, we used a similar method to that previously published [21–23] with modification; a detailed description follows. The patient’s clinical description was translated to HPO terms using Doc2Hpo [24] and manually verified. HPO encoded phenotypes are available for known disease genes through Online Mendelian Inheritance in Man (OMIM.org) [25]. HPO encoded phenotypes for known diseases were extracted from OMIM and Orphanet. Using the ontologyX suite of R packages [26], a pairwise Lin semantic similarity score [27] was calculated between the patient’s HPO term set and the HPO term sets of all known genes encompassed by proband *dn*CNVs. To assess for multilocus pathogenic variation, the phenotypic similarity score of the proband was compared to the combined phenotype associated with sets of known disease-associated genes encompassed by *dn*CNVs. Due to the limited sample size, a cutoff of 5% was arbitrarily selected to aid in the determination of multilocus pathogenic variation in conjunction with phenotypic overlap assessed by grid comparison of proband and disease gene-associated phenotypes.

## Results

### Ascertainment and identification of a new individual with genomic M*dn*CNV phenotype

**Fig. 1:**
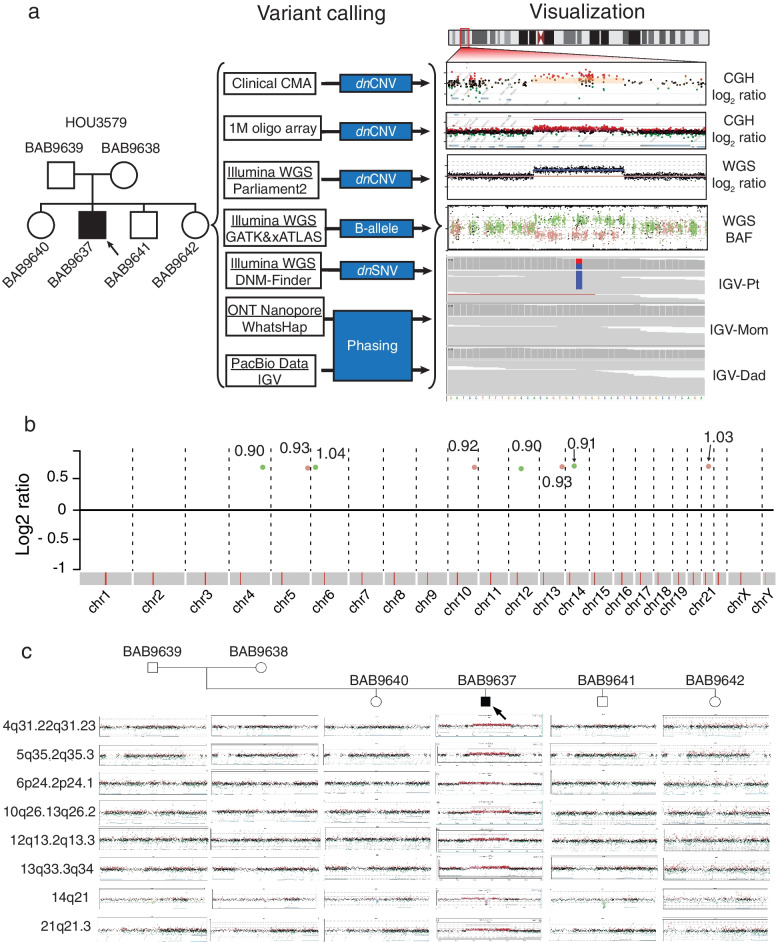
*dn*CNV and *dn*SNV identified with multiple genomic approaches. **a** Pedigree (left) of the M*dn*CNV family HOU3579. In the middle, the sequencing platform and variant calling pipeline are illustrated. Shown on the right, from top to bottom, is  the visualization of an example of *dn*CNV in CMA, 1 M aCGH, short-read genome sequencing read depth, short-read genome sequencing B-allele frequency, and IGV view a high-quality *dn*SNV call. **b** Log2 ratio of phased *dn*CNV in genome-wide view with chromosomes along the *x*-axis. Gains present on chromosomes 4, 6, 12, and 14 are each indicated with a green dot representing duplication on the paternally inherited chromosome. Gains present on chromosomes 5, 10, 13, and 21 are each indicated with a pink dot representing duplication on the maternally inherited chromosome. The text adjacent to each dot denotes the size (in Mb) of each *dn*CNV. **c** Pedigree of M*dn*CNV family (top) with aCGH result for each *dn*CNV region. Parental origin of each chromosome harboring a *dn*CNV in the proband is indicated by a “P” (paternal) or “M” (maternal) on each array

Since the identification of the five M*dn*CNV families at Baylor Genetics (BG) [4], another individual (BAB9637) with potential M*dn*CNV was identified at BG. Clinical CMA performed on proband DNA revealed seven large, ~ 1 Mb, rare variant copy number gains mapping to seven different chromosomes. In addition, an apparent 7 Mb absence of heterozygosity (AOH) genomic region was observed in exome sequencing data mapping to chromosome 15q14q21.1. To capture the full spectrum of *dn*CNVs, the subject, siblings, and parents’ DNA was analyzed further in the research setting using a high-resolution aCGH. Short- and long-read genome sequencing was performed on proband and parental DNA with an average depth of coverage of 35 × and 25 × , respectively. This multimodal genomic analysis approach (Fig. 1a, b, Additional file 1: Table S3) demonstrated eight tandem duplications, confirming the seven duplications previously identified on clinical CMA and revealing an eighth duplication not previously detected. The eight duplications mapped to different chromosomes and showed their sizes ranged from 899.1 to 1041.6 kb, i.e., ~ 1 Mb.

The high-resolution aCGH and breakpoint PCR confirmed that these eight duplications were only present in the proband (Fig. 1c and Additional file 1: Figures S1, S2). However, the apparent 7 Mb AOH region observed in chr15q14q21.1 by clinical CMA was not supported by the B-allele frequency calculated from genome sequencing data. This is likely due to genome sequencing interrogating a higher density of SNP sites (*n* = 9080) compared to clinical CMA (*n* = 50) (Additional file 1: Figure S3). An apparently homozygous ~ 60.2 kb deletion was identified within a rare *de novo *duplication on chromosome 14. Both parents are heterozygous for the deletion. The allele frequency of the deletion in the control population is 0.139 based on an allele count of 2990/21518 alleles in the gnomAD SV v2.1 database. The deletion allele frequency ranges from 0.043 in the African population to 0.339 in the Latino population (Additional file 1: Figure S4). We explored the potential association between both replication time and CNV regions by overlapping the duplication region to the replication time map of four embryonic stem cell lines [28]. Our analysis did not suggest a preference of *dn*CNV occurring regarding replication timing, i.e., *dn*CNVs were mapped to either late or early replication regions. Only microhomologies were found at the breakpoint junction for five *dn*CNVs (Additional file 1: Figure S1) which suggests a replicative instead of homologous recombination-based mechanism for the *dn*CNV formation in this case.

**Fig. 2:**
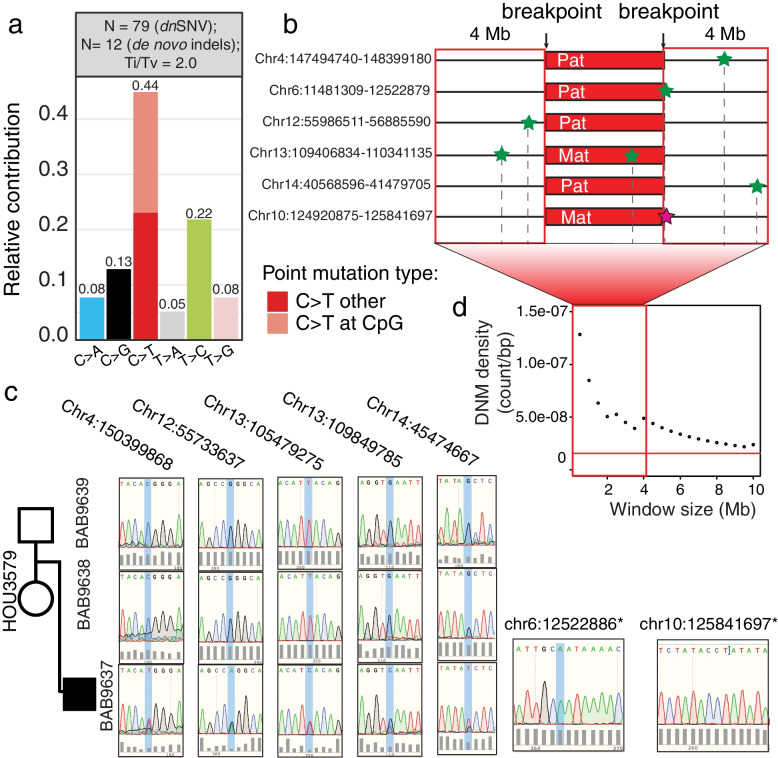
*De novo* variants detected in BAB9637. **a** Ratio of transition to transversion is shown at the top of the bar graph. The bar graph represents the relative contribution of types of SNV. **b** Horizontal red bars represent each *dn*CNV that is associated with DNMs in proximity to the breakpoint. All seven DNMs found within 4 Mb of the breakpoints are highlighted with a star at the relative location. Maternal and paternal DNMs are highlighted in pink and green, respectively. The DNM at chr4, chr6, chr12, chr10, and chr14 are *in cis* with *dn*CNVs. **c** Sanger traces are visualized (proband, mother, and father) for DNMs. * The variant at the breakpoint junction. **d** Density of unphased DNM corresponding to the window size highlighting continuous drop-off of observed DNM after 4 Mb

With SR sequencing data, we identified 91 DNMs including 79 SNVs (transition to transversion ratio [Ti: Tv] = 2.0) and 12 indels (Fig. 2 and Additional file 3: Table S4). By combining short- and long-read sequencing data, we were able to phase 50 *dn*SNVs, of which 80% were of paternal origin (Additional file 3: Table S4). The predominance of paternal inheritance of *dn*SNVs was anticipated due to the accumulation of variants in aging male gametes [29, 30]. The genomic mapping of *dn*SNVs on the haploid human genome reference did not show any obvious clustering of variants. We calculated the distance of DNM to the nearest *dn*CNV breakpoint and found 7 (8.2%) DNMs within 4 Mb of the nearest breakpoint (Additional file 3: Table S4 and Fig. 2). The density of DNMs within 4 Mb of the breakpoint junctions was significantly higher than expected based on the number of DNMs and size of the genome (Additional file 1: Table S5, Fig. 2d) and was also significantly higher than the density of DNMs at the same genomic location in approximately 3000 control genomes [18]. We considered only the phased DNMs *in cis* with *dn*CNVs, which revealed that the observed density of DNMs was significantly higher than expected for either the maternal or paternal inherited haploid genome consistent with the hypermutation hypothesis [14, 31] of an underlying error-prone repair mechanism, microhomology-mediated break-induced replication (MMBIR) producing both CNVs and local SNVs (Additional file 1: Table S5).

### Elevated post-zygotic *de novo* substitution rate in M*dn*CNV families

**Fig. 3:**
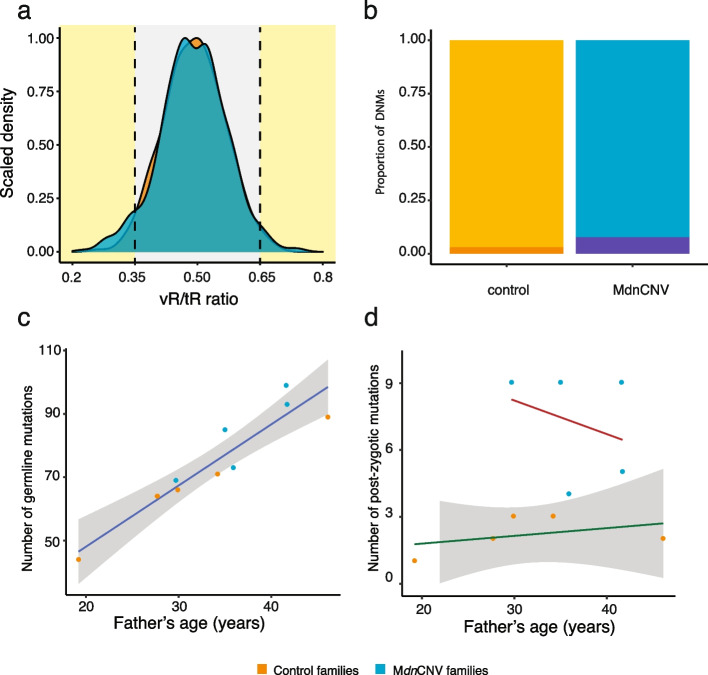
The number and mutational pattern of pre-zygotic and post-zygotic *de novo* mutations in M*dn*CNV families (blue) versus controls (orange). **a** The VAF distribution of *de novo* substitutions in M*dn*CNV (blue) and control (orange) families. **b** The proportion of DNMs that are predicted to be post-zygotic mutations (dark orange/blue). **c** The number of pre-zygotic (germline) mutations is positively correlated with paternal age. The gray area denotes the region covered by the 95% confidence interval of the slope and intercept of the linear regression lines. **d** The number of post-zygotic mutations shows no correlation with age

In addition to the new M*dn*CNV family (BAB9637), four previously reported M*dn*CNV families (BAB3097, BAB3596, mCNV3/BAB9484, and mCNV7, Table 1) were genome sequenced with an average depth of coverage of 40 × . Through clinical array analysis, five anonymized families ascertained without M*dn*CNV were included as controls. We identified 470 (transition to transversion ratio [Ti:Tv] = 2.2, 95% confidence interval (CI) = 1.62–2.30) and 361 ([Ti:Tv] = 2.2, CI = 1.29–3.1) high-confidence *de novo* substitutions in M*dn*CNV and control families, respectively. SNV substitution mutations that occur during early development can lead to mosaicism in peripheral blood leukocyte-derived DNA, which will change the expectation of variant allele fractions (VAFs), e.g., somatic mutation arising during first cell division will have expected VAFs of 25%. The *de novo* substitutions were classified into potential germline mutation, or post-zygotic mutation based on the variant read to total read ratio (Fig. 3a, b, and “Methods”). The number of potential germline substitutions in M*dn*CNV families appears to be comparable (*p* = 0.1, F-test) to control families with an average paternal age effect of 1.9 (95% confidence interval 1.32–2.54) (Fig. 3c, d). However, the proportion of potential post-zygotic substitutions appears to be higher (*z*-test, *p* = 0.004) in M*dn*CNV (8.0%, *n* = 36) versus control families (3.2%, *n* = 11). The paternal age effect on the number of potential post-zygotic variants was not significant for either group.

**Table 1:** Sample information and *de novo* substitution calls

		**M*dn*CNV cases**						**Anonymized control cases**					
**Family**		**HOU1209**^**a**^	**HOU1404**^**a**^	**HOU3425**^**a**^	**HOU3579**	**mCNV7**^**a**^	**Average (95 CI)**	**Fam1**	**Fam2**	**Fam3**	**Fam4**	**Fam5**	**Average (95 CI)**
**Individual (gender)**		**BAB3097 (F)**	**BAB3596 (M)**	**mCNV3/BAB9484 (M)**	**BAB9637 (M)**	**mCNV7 (F)**		**P1**	**P2**	**P3**	**P4**	**P5**	
Parental age of conception (years)	Mother	29.4	37.0	37.8	36.9	35	35.2 (31.0–39.5)	17.2	30.3	41	28.4	32.3	29.8 (19.2–40.5)
	Father	29.7	41.7	41.6	35.9	37	37.2 (31.0–43.3)	19.2	29.9	46.1	27.7	34.2	31.4 (19.2–43.7)
Number of *de novo* substitution mutations^b^	Germline SNV	75	94	102	74	89	93.2 (75.3–111.1)	47	69	98	64	72	72.2 (48.8–95)
	Post-zygotic SNV	9	5	9	4	9	7.2 (4.1–10.3)**	1	3	2	2	3	2.2 (1.2–3.2)
	Total	84	99	111	78	98	93.2 (75.3–111.0)	48	72	100	66	75	72.2 (48.9–95.5)
	Ti:Tv ratio	1.8	2.1	1.6	2.0	2.3	2.2 (1.62–2.30)	2.4	2.4	1.1	3.1	2.0	2.2 (1.29–3.1)

*M* Male, *F* Female, *SNV* Single-nucleotide variant, *CNV* Copy number variant, *CI* Confidence interval, **p < 0.01; *Ti* Transition, *Tv* Transversion

^a^M*dn*CNV families first reported in Liu et al.[9]

^b^Variants at the breakpoint junction are not included for the comparison

### Mutation pattern analysis for DNMs

We cataloged 466 *de novo* substitutions into seven different mutation types, including the six possible single base-pair substitutions and one category representing CpG deamination. The mutation pattern of germline substitutions in M*dn*CNV families does not appear to be different from controls, with ~ 40% of the germline mutations being C > T transition variants (Additional file 1: Figure S5). This is consistent with the elevated rate of spontaneous deamination of 5-methyl-cytosine, which occurs at CpG dinucleotides [32].

**Fig. 4:**
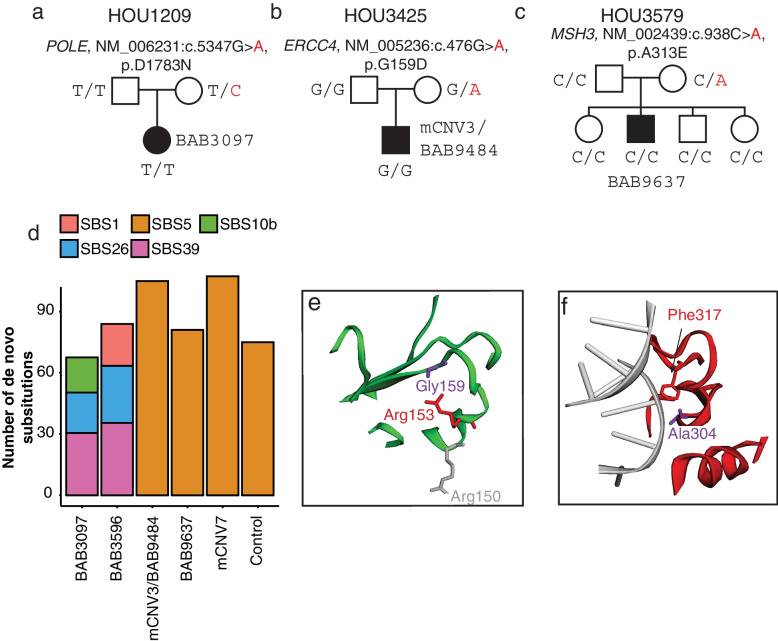
Maternal variants potentially contributing to genome instability. **a–c** M*dn*CNV pedigrees with identified rare VUS maternal variants affecting genes involved in DNA repair or replicaion. **d** Bar plot shows the contribution of SBS signatures refitted by on genome-wide *dn*SNV. Predicted protein structure plots show the amino acid change in proximity to previously reported variants in protein ERCC4 (**e**) and MSH3 (**f**). Molecular modeling images were acquired from Varsite [33], with pathogenetic variants from ClinVar mapped. The amino acid residues in red reveal the change caused by variants reported here and the ones in purple or gray reveal the reported pathogenetic variants from ClinVar

A higher-resolution mutation substitution pattern analysis was performed on genome-wide *dn*SNVs to investigate the contribution of validated mutational signatures extracted from somatic mutation analysis of the cancer genome [12]. We found that SBS5 and SBS1 explain the majority of genome-wide *dn*SNVs in all the control and three of M*dn*CNV probands (BAB9484, BAB9637, and mCNV7, Fig. 4d, Additional file 2: Table S2). In contrast, the other mutational signatures (SBS10b, SBS26, and SBS39) were observed in two M*dn*CNV cases (BAB3097 and BAB3596) (Fig. 4d). To exclude potential context bias around the *dn*CNV region, e.g., C > T at CpG for TCG context, mutation signatures were reassessed on *dn*SNV not present within *dn*CNV and 1 Mb distance flanking the breakpoints. After reassessment, SBS1, SBS26, and SBS39 are still associated with BAB3596. SBS39, SBS10b, and SBS37 instead of SBS39, SBS10b, and SBSB26 are associated with BAB3097 (Fig. 4d and Additional file 1: Figure S6), which suggests a nonspecific association of SBS26 with BAB3097.

Rare variant analyses in the maternal genome identified deleterious variants in three genes involving DNA repair pathways from three M*dn*CNV families (HOU1209, HOU3425, and HOU3579, Fig. 4a, b and c). The *POLE* variant (NM_006231:exon39:c.5347G > A:p.D1783N) identified in HOU1209 family (Fig. 4a) is ultrarare in a population database (rs149893630, gnomAD 0.001%). In silico analysis supports that this missense variant has a deleterious effect on protein structure/function (Polyphen2: Probably Damaging; SIFT: Deleterious; CADD v1.6 = 29.4). We also identified variants in *ERCC4* and *MSH3* from two other M*dn*CNV families (HOU3425 and HOU3579, Fig. 4b,c). Both variants (*ERCC4*:NM_005236:exon3:c.476G > A:p.G159D and *MSH3*:NM_002439:exon6:c.938C > A:p.A313E) are absent from the gnomAD database and are predicted to be pathogenic (Polyphen2: Damaging; Damaging; SIFT: Deleterious, Deleterious; CADD v1.6 = 29.5 and 33.0, respectively). The amino acid residue at both sites are highly conserved from the 161-aligned and 144-aligned protein sequence based on predictions from the tool Varsite [33]. The mutated residues are in proximity to the reported sequences with predicted functional consequences based on 3D protein models (Fig. 4e,f) from Varsite [33] and MichelaNglo [34].

### Phenotypic variability derived from variable regions affected by *dn*CNV in the M*dn*CNV cohort

While probands described with the M*dn*CNV phenomenon have a similar molecular finding on analysis for CNV in their genomes, their phenotypic features observed at clinical evaluation are variable. Within the cohort of five individuals with M*dn*CNV phenotype, three patients have detailed clinical information available (Supplementary text) for genotype and phenotype analysis. To discern whether phenotypic variability results from the variable regions of the genome affected by the M*dn*CNV phenomenon, HPO-based evaluation was also performed on the three M*dn*CNV probands (BAB9637, mCNV3/BAB9484, and BAB3097).

**Fig. 5:**
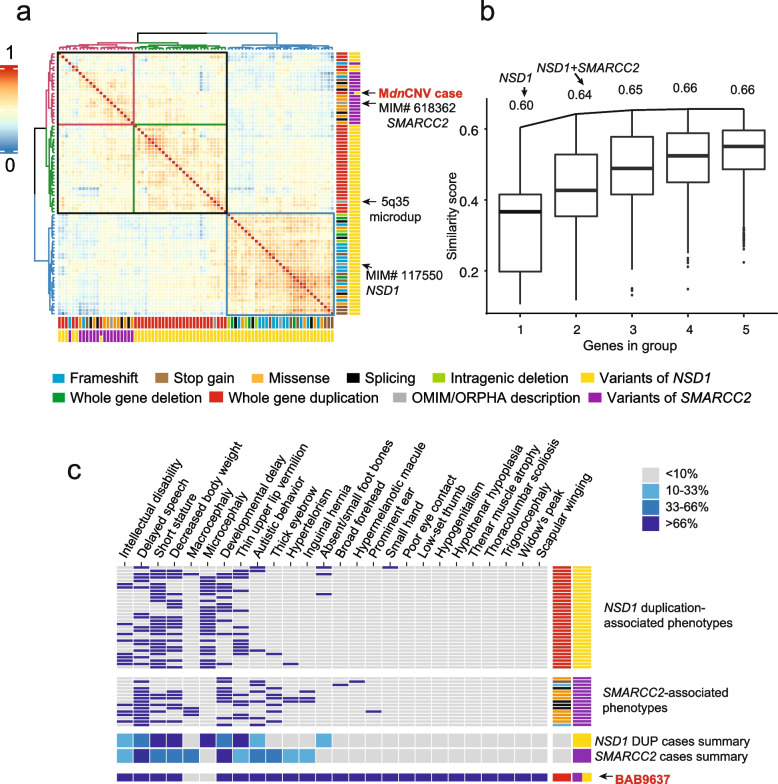
Phenotype similarity score analysis for disease-associated genes and potential gene combinations for the multiple pathogenic variant case (BAB9637). **a** Heatmap representing color-coded Lin semantic similarity scores of BAB9637 and database annotated phenotypes included. Both rows and columns are clustered using pairwise similarity scores and the Ward's method. The dendrogram is present at the top and to the left of the heatmap. Colored columns are depicted at the bottom and to the right and annotate variant type and affected gene as defined at the bottom. **b** Distribution of similarity scores in each known disease-associated gene group with *n* = 1 to *n* = 5 genes. A black line connects the max score of subsequent subsets of groups, e.g., max score of groups with one gene to max score of groups with two genes. **c** Annotation grid demonstrates individual reported *NSD1* duplication proband phenotypes, individual *SMARCC2* LoF proband phenotypes, *NSD1* DUP and *SMARCC2*-associated clinical phenotypes summary, and proband phenotypes for BAB9637. From left to right aligned HPO phenotype, blue squares indicate the presence of the phenotype, i.e., HPO term, while gray represents the absence of the term. The clinical phenotype summary was based on the cases used in HPO analysis, with the degree of shading indicating the percent of reported cases shown here for which a particular feature has been observed, as defined in the legend in the top right corner

Concerning BAB9637, we classified the *de novo* duplication encompassing *NSD1* associated with Sotos syndrome (MIM#117550) as a pathogenic variant, based on the most recent consensus recommendation of the American College of Medical Genetics and Genomics (ACMG) (Table 2) [35]. The remaining *dn*CNVs (7/8) were classified as variants of unknown significance (VUS). To investigate the contribution of the other *dn*CNV encompassing genes to patient phenotype, we performed Lin semantic similarity analysis of BAB9637’s phenotype to OMIM/Orphanet HPO annotated gene and disease-associated phenotypes. The Lin semantic similarity scores [27] suggest that, of the genes encompassed by *dn*CNV regions, *NSD1* and *SMARCC2* were the two with the highest phenotypic similarity score (0.60 and 0.59, respectively) to the proband’s phenotype (Additional file 1: Table S6). We compared the phenotype similarity of BAB9637 with previously reported probands with only *NSD1* variants (30 probands with *NSD1* duplication, 30 probands with *NSD1* deletion/LoF variant) or only *SMARCC2* variants. Three clusters were observed, one consisting of all 30 included *NSD1* deletion/LoF probands, the second of 25/30 (81%) of reported *NSD1* duplication probands, and the third containing 16/16 *SMARCC2* LoF probands, 5/30 of *NSD1* duplication probands, and BAB9637 (Fig. 5a).

**Table 2:** Genes mapping to the duplicated genomic region

Sample	Locus	*dn*CNV Coordinates(GRCh38)	*dn*CNV type	OMIM disease genes (associated trait MIM#) mapping in duplicated region	Number of genes(pLi > 0.9)	Total number of genes overlapping duplication region	ACMG classification evidence*	ACMG classification
BAB9637	4q31.22q31.23	chr4:147494740-148399180	DUP	*EDNRA* (#616367, AD); *NR3C2 *(#177735, AD)	2	10	1A, 2J, 3A, 4D	VUS
BAB9637	5q35.2q35.3	chr5:176449583-177376826	DUP	*SNCB* (#127750, AD); *NSD1* (#117550, AD)	3	23	1A, 2A	Pathogenic
BAB9637	6p24.2p24.1	chr6:11481309-12522879	DUP	*EDN1* (#612798, AD; #615706, AR)	1	10	1A, 3A, 4D	VUS
BAB9637	10q26.13q26.2	chr10:124920875-125841697	DUP	*UROS* (#263700, AR); *MMP21 *(#616749, AR)	1	16	1A, 3A, 4D	VUS
BAB9637	12q13.2q13.3	chr12:55986511-56885590	DUP	*MIP *(#615274, AD), *ERBB3 *(#133180, AD; #607598, AR),*SMARCC2 *(#618362, AD), *RPS26 (*#613309, AD*), SLC39A5 *(#615946, AD), *SUOX *(#272300, AR), *STAT2 *(#618886, AR; #616636, AR)	8	40	1A, 2H, 3B, 4B	VUS
BAB9637	13q33.3q34	chr13:109406834-110341135	DUP	*COL4A1 *(#180000, AD; #611773, AD; #175780, AD; #618564, AD), *COL4A2 *(#614483, AD), *IRS2* (#125853, AD)	1	8	1A, 3A, 4D	VUS
BAB9637	14q21.1	chr14:40568596-41479705	DUP		0	0	1B	VUS
BAB9637	21q21.3	chr21:28158347-29192300	DUP		1	12	1A, 3A, 4D	VUS
BAB3097	1p36.22p36.13	chr1:10115497-16283149	DUP	*KIF1B *(#118210, AD; #171300, AD; #256700), *PEX14 *(#614887, AR),* TARDBP *(#612069, AD)*, MASP2 *(#613791, AR), *MTOR *(#616638, AD), *UBIAD1 *(#121800, AD), *MAD2L2 *(#617243, AR), *MTHFR *(#236250, AR), *CLCN6 *(#619173, AD), *NPPA *(#612201, AD; #615745, AR), *PLOD1 *(#225400, AR), *MFN2 *(#609260, AD; #617087, AR; #601152, AD), *VPS13D *(#607317, AR), *CELA2A *(#618620, AD), *SPEN *(#619312, AD), *CLCNKA *(#613090, DR), *CLCNKB *(#607364, AR; #613090, DR), *EPHA2 *(#116600, AD)	13	167	1A, 3C, 4C	Likely Pathogenic
BAB3097	3q13.33q21.1	chr3:122157406-123113479	DUP	*CASR *(#239200, AD/AR; #601198, AD; #145980, AD),* CSTA *(#607936, AR)	19	2	1A, 3A, 4D	VUS
BAB3097	5p12	chr5:44375961-44815730	DUP	*FGF10 *(#180920, AD; #149730, AD)	0	2	1A, 2J, 3A, 4D	VUS
BAB3097	5q33.3q34	chr5:158887731-164722046	DUP	*IL12B *(#614890, AR), *GABRB2 *(#617829, AD), *GABRA1 *(#615744, AD), *GABRG2 *(#618396, AD; #607681, AD)	5	42	1A, 3A, 4D	VUS
BAB3097	9p13.3	chr9:33492358-34725916	TRP	*UBAP1* (#618418, AD), *MYORG *(#618317, AR), *DNAI1 *(#244400, AR), *SIGMAR1 *(#614373, AR; #605726, AR), *GALT *(#230400, AR), *IL11RA *(#614188, AR)	4	53	1A, 3C, 4D	VUS
BAB3097	17p12p11.2	chr17:11915997-17892664	DUP	*DNAH9 *(#618300, AR), *MYOCD* (#618719, AD), *ELAC2 *(#615440, AR), *COX10 *(#619046, AR), *PMP22* (#139393, AD; #118220, AD; #118300 AD; #145900, AD; #162500, AD; #180800, AD), *TTC19 *(#615157, AR), *PIGL *(#280000, AR), *TNFRSF13B *(#240500, AD/AR), *FLCN *(#135150, AD; #173600, AD), *RAI1 *(#182290, AD), *SREBF1 *(#619016, AD; #158310, AD)	7	110	1A, 2A	Pathogenic
BAB3097	22q13.31p13.32	chr22:47979382-48288823	DUP		0	1	1A, 3A, 4D	VUS
mCNV3/BAB9484	1p31.3p31.1	chr1:66885559-77949895	DUP	*SLC35D1 *(#269250, AR), *WLS *(#619648, AR), *RPE65 *(#204100, AR; #613794, AR; #618697, AD), *CTH* (#219500, AR), *TNNI3K *(#616117, AD), *ACADM* (#201450, AR), *PIGK* (#618879, AR), *NEXN *(#613122, AD; #613876, AD)	9	108	1A, 3C, 4D	VUS
mCNV3/BAB9484	1q42.2q42.3	chr1:233450789-235471180	DUP	*COA6 *(#616501, AR), *IRF2BP2* (#617765, AD), *GGPS1 *(#619518, AR), *TBCE *(#617207, AR; #241410, AR; #244460, AR), *B3GALNT2 *(#615181, AR)	1	35	1A, 3B, 4D	VUS
mCNV3/BAB9484	2p13.3	chr2:69512973-71153026	DUP	*ASPRV1 *(#146750, AD), *TIA1 *(#619133, AD; #604454, AD/AR), *FIGLA *(#612310, AD), *ATP6V1B1* (#267300, AR), *MCEE *(#251120, AR)	2	44	1A, 3B, 4D	VUS
mCNV3/BAB9484	3q26.32	chr3:176661565-177473432	DUP	*TBL1XR1 *(#616944, AD; #602342, AD)	1	8	1A, 2A, 3A, 4C	Likely Pathogenic
mCNV3/BAB9484	9q22.2	chr9:89241202-90787598	DUP	*SECISBP2 *(#609698, AR)	2	38	1A, 3B, 4D	VUS
mCNV3/BAB9484	11p12p11.2	chr11:42871836-44852545	DUP	*EXT2 *(#133701, AD; #616682, AR), *ALX4 *(#613451, AR; #609597, AD; #615529, AD)	2	25	1A, 2H, 3B, 4D	VUS
mCNV3/BAB9484	16q22.2	chr16:71219688-71768356	DUP	*HYDIN *(#608647, AR), *TAT* (#276600, AR), *AP1G1 *(#619467, AD)	1	16	1A, 3A, 4D	VUS
mCNV3/BAB9484	20q13.33	chr20:61800345-63644611	DUP	*OSBPL2 *(#616340, AD)*, GATA5 *(#617912, AD/AR), *COL9A3 *(#600969, AD), *SLC17A9 *(#616063, AD), *CHRNA4 *(#600513, AD), *KCNQ2 *(#613720, AD; #121200, AD)*, EEF1A2 *(#616409, AD; #616393, AD)	4	15	1A, 3A, 4D	VUS

*pLI* probability of being Loss‐of‐function Intolerant, *AD* autosomal dominant, *AR* autosomal recessive, *DR* digenic recessive, *ACMG* American College of Medical Genetics and Genomics, *OMIM* Online Mendelian Inheritance in Man. *Evidence code based on ACMG consensus recommendation; *DUP* duplication, *TRP* Triplication

We evaluated whether the observed phenotypes of BAB9637 are better explained by a combinatorial effect of two or more affected genes, i.e., dual molecular diagnosis or MPV, multilocus pathogenic variation. The combination of *NSD1* and *SMARCC2* yielded the highest similarity score (0.64) among all pairwise gene combinations (Fig. 5b). Compared with a single gene model, a dual molecular diagnosis model improved the phenotypic similarity score by > 5%. The increase in phenotypic similarity score was not substantial in the 3rd, the 4th, or the 5th gene (~ 1%) compared to the dual molecular diagnosis model (Additional file 1: Table S6). A review of the phenotypes associated with *NSD1* and *SMARCC2* in grid format reveals that their phenotypic spectrums largely overlap (Fig. 5c). The grid visualization also highlights the presence of phenotypes in the proband that has predominantly or only been observed in *NSD1* probands (e.g., absent/small foot bones) or in *SMARCC2* probands (e.g., thick eyebrows, inguinal hernia; Fig. 5c). The combination of phenotypic contributions from defects caused by both *NSD1* and *SMARCC2*, therefore, offers the most parsimonious explanation to account for the overall phenotypic presentation in the patient, supporting a multilocus pathogenic variation (MPV) model of blended phenotypic traits caused by perturbation of genes at both loci to constitute the proband’s phenotype.

For the BAB3097 M*dn*CNV case, two *de novo* duplication CNV, one (17p12p11.2) with two genes within a contiguous duplication, *RAI1* and *PMP22* associated with Yuan-Harel-Lupski Syndrome (YUHAL; MIM#616652) [36], and the other (1p36.22p36.13) encompassing established haploinsufficiency gene *SPEN*, meet ACMG criteria for pathogenicity (pathogenic/likely pathogenic) and appear to be the clinically relevant genes within these CNV intervals (Table 2). *RAI1* has the highest similarity score (Lin similarity score 0.52), and *PMP22* ranks 11 (Lin similarity score 0.39) among *dn*CNV encompassed genes (Additional file 1: Table S6). The proband phenotype was analyzed for phenotypic similarity with 17 reported YUHAL cases and OMIM diseases associated with either *RAI1* or *PMP22*. Three clusters were observed, one consisting of 12/17 YUHAL probands, the second of 5/17 of reported YUHAL probands and BAB3097, and the last one containing *PMP22* associated neurological diseases (Additional file 1: Figure S7a). The phenotypic grid suggests 7/11 of BAB3097’s observed phenotype matches with YUHAL associated phenotypes reported in Yuan et al. (Additional file 1: Figure S7b) [36]. Additional genes with a high phenotypic similarity score include *FLCN* (Lin similarity score 0.50, ranked second) located at 17p12p11.2 and *SPEN* (Lin similarity score 0.45, ranked fourth) at 1p36.22p36.13 (Additional file 1: Table S6). Among all dual molecular diagnosis combinations, 13 have more than 5% improvement of similarity scores over single gene similarity scores. Of those 13 combinations, all have at least one gene within the two regions (17p12p11.2 and 1p36.22p36.13), and 8/13 are made up exclusively of genes within the same regions (Additional file 1: Table S6).

For mCNV3/BAB9484, we classify the *de novo* duplication at 3q26.32 as likely pathogenic based on ACMG guidelines (Table 2). *TBL1XR1* was the only coding gene at the duplication region and had the highest phenotypic similarity score (Lin semantic similarity score = 0.50) among all *dn*CNV encompassed genes. The second highest gene is *EXT2* (Lin similarity score = 0.39). Of all dual molecular diagnosis combinations, three have more than 5% improvement of similarity score over a single gene (Additional file 1: Table S6). The phenotype grid suggests 3/10 exact match of *TBL1XR1* associated terms, including hypertelorism, brachycephaly, and wide intermammillary distance (Additional file 1: Figure S8).

## Discussion

Our study utilized multimodal genomic approaches to investigate *de novo* genomic rearrangements in an M*dn*CNV family, characterize the mutational event that instigated M*dn*CNV across the genome, identify *de novo* SNV genome-wide, phase CNV and SNV haplotypes, and explore potential driver genes contributing to the observed clinical phenotype. Accurately capturing structural variants and complexities generated during CNV mutagenesis, including novel DNA rearrangement junctions or DNA break/join-points and other SNV mutational signatures of CNV mutagenesis, is challenging given the length limit of sequencing reads in SR genome sequencing. Genome-wide ascertainment of *dn*CNVs, breakpoint junction sequence, and *dn*SNVs were enabled by implementing both LR and SR genome sequencing. LR sequencing, either PacBio [14] or Nanopore as shown here, allows direct phasing of variants multiple kilobases apart, which in turn allows direct observation of *dn*SNV *in cis* with breakpoint junctions.

The average size of tandem duplication observed in the newly identified M*dn*CNV individual is around 1 Mb, consistent with the observation in the five reported M*dn*CNV individuals [4]. The size is also within a similar size range “window” of replication domains [37] and topologically associating domains (TAD) [38]. Recent study suggests the replication domain boundary is associated with TAD [39]. The size of tandem duplication can be limited within the boundary of the genome organization, e.g., the three-dimensional genome folding and accessibility of DNA during zygotic development. Further analysis on the dynamics of chromatin architecture during zygote development is warranted to address potential influence or constraints on rearrangement size.

We postulate that the CNV generating mechanism could be MMBIR, based on the shared characteristics of microhomology/microhomeology at breakpoint junctions, complexity in the form of associated indels, and the increased occurrence of base substitutions and indels within 4 Mb of junctions. MMBIR, a form of break-induced replication, occurs when a replication fork has collapsed/stalled and restarted. Alternatively, the unrepaired DNA nicks could result in a collapsed fork that is resolved by a mixed NHEJ/MMBIR mechanism, e.g., restart-bypass [40]. Regardless of which mechanism(s) are involved, we suggest that there were multiple broken replication forks in a single zygotic cell and that the M*dn*CNV event, therefore, may have resulted from cell-wide replication stress, such as energy or substrate unavailability that resulted in multiple broken replication forks.

We found that the mutation rate for *dn*CNV and substitution was elevated in five M*dn*CNV families. The post-zygotic mutational events happened at the early development stage, e.g., first cell division has a VAF of 10–35% [41]. The mutation rate during early embryonic development is higher than in germline cells, expecting 2–3 substitutions per generation [41]. The post-zygotic substitution rate in M*dn*CNV families is three times higher than the rate in control (*z*-test, *p* = 0.004, Table 1). The mechanism(s) causing the CNV mutator phenotype is thought to be due to a transient mutagenesis event restricted to the peri-zygotic stage of development [4]. We suspect the mechanism(s) could also lead to SNV hypermutation limited to the peri-zygotic period. However, high-depth genome sequencing is required for the estimation of mosaicism accurately and further supports this hypothesis.

The *dn*SNV mutational pattern and rare variant analysis suggest multiple mechanisms contributing to the transient peri-zygotic genome instability causing the M*dn*CNV phenomenon. One of the mechanisms could be that non-inherited variants in the maternal genome affect zygotic genome integrity, whereby maternal mRNA stored in the oocyte impacts the first few cell divisions during embryonic development. Rare variant analyses identified maternal variants in three DNA repair genes *POLE*, *ERCC4*, and *MSH3* with predicted deleterious effects on the function that could contribute to peri-zygotic genome instability. The gene *POLE* encodes the catalytic subunit of polymerase *ε* which plays a major role in the DNA replication [42]. The *POLE* variant identified in family HOU1209 is located in the C-terminal structure of the subunit which is essential for replisome assembly and checkpoint activation [43, 44]. The maternal variants identified in family HOU3425 (*ERCC4*, NM_005236:c.476G > A, p.G159D) and HOU3579 (*MSH3*, NM_002439:c.938C > A, p.A313E) may contribute to replication stress through different mechanisms. *ERCC4* (MIM#133520, also known as XPF) encodes the endonuclease catalytic subunit that is involved in nucleotide excision repair (NER) and removes DNA interstrand crosslinking damage [45, 46]. The interstrand crosslink can prevent DNA strand separation and physically block DNA replication and transcription, leading to genomic instability. The mutated residue (*ERCC4*, NM_005236:c.476G > A, p.G159D) is near two reported functional residues that are relevant to the crosslink repair activity of ERCC4 (Fig. 4e). The nearest one (rs121913050, p.R153P), located 2.2 Å away from the mutated residue, is classified as pathogenic in ClinVar and causes XFE progeroid syndrome (MIM#610965) in a homozygous state [47, 48]. The second variant (rs145402255, p.R150C), located 10.8 Å away from the mutant residue is classified as likely pathogenic in ClinVar. In vitro cell modeling suggests the variant (rs145402255, p.R150C) can mildly disrupt the interstrand crosslink repair activity [49]. *MSH3* encodes a protein that forms a heterodimer with *MSH2* that is responsible for mismatch repair (MMR) and double-stranded DNA repair [50, 51]. In summary, these variants may represent genetic modifiers rather than causative drivers that may contribute to the M*dn*CNV phenomenon. Additionally, 4/5 M*dn*CNV families (Table 1) were of advanced parental age (> 35 years old) at the time of conception, which may have reduced the capacity of DNA repair in oocytes and therefore have a contributory effect on the genomic stability of the zygote.

Mutational signature analysis can be used to decipher the potential mutational processes underlying individual cancer or germline hypermutation [52, 53]. A recent study has revealed genetic and environmental contributions to the germline *dn*SNV hypermutation [53]. Our study explored the utility of mutational signature analysis to decode potential mutational processes in the context of *dn*CNV hypermutation. However, further studies are still warranted to understand the mutational process(s) during the peri-zygotic development stage. Mutational signature analysis on embryonic somatic mutations (SNVs and CNVs) may help solve the puzzle. Additionally, further studies are needed to confirm mutational signatures within certain genes or allele-specific effects.

Reciprocal copy number changes in dosage-sensitive gene loci can manifest traits on the opposite ends of a phenotypic spectrum, a phenomenon known as mirror traits [54]. The quantitative trait for head size, or occipital frontal circumference (OFC), illustrates this concept: reciprocal CNVs at multiple loci can drive mirror trait expression manifesting as large (macrocephaly) or small (microcephaly) head size [54–56]. Semantic similarity analysis using HPO terms can quantify the similarity of matching phenotype, therefore objectively teasing out the contribution of genes within *dn*CNV regions. The similarity score correctly ranked the established genes in the *dn*CNV region and suggested additional genes from the other regions contributing to two of the M*dn*CNV cases. Quantitative analyses of the clinical phenotypes in BAB9637 versus known disease genes mapping within the duplications implicate *NSD1* as a triplosensitivity trait locus that contributes to the clinical phenotype observed. The 5q35 microdeletion causing haploinsufficiency of *NSD1* has been associated with Sotos syndrome (MIM#117550). The characteristic clinical features of Sotos syndrome include overgrowth, characteristic facial dysmorphisms, intellectual disability (ID), developmental delay (DD), and macrocephaly. The proband (BAB9637) has overlapping features, including DD and ID. However, regarding the quantitative traits of head circumference and height, the proband (lower-than-average head circumference [*Z* =  − 0.62]; short stature [*Z* =  − 3.01]) with a duplication encompassing *NSD1* lies on the opposite end of the phenotypic spectrum compared to individuals with a diagnosis of Sotos syndrome. Furthermore, the clinical presentation of our proband, which includes DD/ID, and short stature, is consistent with that of 31 affected individuals previously reported across multiple studies [57] to have a *de novo* or familial duplication of the genomic region whose deletion is commonly associated with the Sotos syndrome (Fig. 5, Additional file 1: Figure S9).

The presence of two highly similar clusters for *NSD1* duplication and *SMARCC2*, and their separation from a more dissimilar *NSD1* deletion/LoF cluster, highlights the shared phenotypic trait between *NSD1* duplication and *SMARCC2* associated phenotypes. The contribution of *NSD1* does not seem to explain all phenotypes observed in the proband, e.g., scoliosis and craniofacial features. Haploinsufficiency of *SMARCC2* has been associated with Coffin-Siris syndrome 8 (MIM#618362). *SMARCC2* duplication is ultrarare, less than 0.0001 in one control study [58], and absent in the personal genomes of neurotypical individuals from population databases (gnomAD v2.1.1). *SMARCC2* duplication has been reported in three cases (size of duplication < 1 Mb) in the *D*atabas*E* of genomi*C* var*I*ation and *P*henotype in *H*umans using *E*nsembl *R*esources (DECIPHER, https://www.deciphergenomics.org/) [59] (Additional file 1: Figure S9). Two genomes with duplications from unrelated patients have a phenotype described, one (DECIPHER patient ID: 343437) in an individual with growth delay and ID, the second duplication (DECIPHER patient ID: 260552) inherited from a parent who has a similar phenotype, including hypertelorism, hypospadias, broad thumb, delayed cranial suture closure, hypotonia, and ID. Data on segregation of duplication variants in DECIPHER individuals was not available.

The potential molecular diagnoses underlying BAB3097’s phenotype highlight another case where *de novo* CNV at more than one locus contributes to disease pathobiology. Contiguous gene duplication encompassing *RAI1* and *PMP22* has been previously described in 17 patients with YUHAL syndrome [36]. Renal phenotypes were observed in 4/17 of patients with *Yu*an-*Ha*rel-*L*upski (YUHAL) syndrome, and while a definitive gene within the duplication interval was not associated with renal phenotypes, *FLCN* was suggested to be a potential contributor. In addition to the duplication at this locus, our analysis suggests duplication at 1p36.22p36.13 may also contribute to the phenotype, with *SPEN* as the potential driver gene. The gene *SPEN* is located at the 1p36 deletion syndrome critical region. Furthermore, truncated variants at *SPEN* suggest the haploinsufficiency of *SPEN* associated with neurodevelopment phenotype, congenital heart defects, and facial dysmorphism [60]. The whole gene duplication of *SPEN* was absent from DGV and gnomAD.

Further study of the phenotypic effect of *SMARCC2* and *SPEN* duplication in more patients, rather than deletion, is necessary to understand dosage sensitivity (i.e., triplosensitivity) at these loci; however, these data in aggregate support the contention that the phenotype of the proband is a blended overlapping phenotype driven by multilocus pathogenic variation (MPV) [21, 61], i.e., duplication of the *NSD1* and *SMARCC2* loci, and their associated traits. Our understanding of emerging concepts, such as which genes or loci are dosage-sensitive, whether haploinsufficiency and triplosensitivity traits will be observed for a given dosage-sensitive gene, and the correlation between dosage-sensitive genes and mirror traits is continuing to evolve with human genetics and genomics studies.

While semantic similarity analysis with patient phenotypes can objectively tease out the contribution of genes within *dn*CNV regions, there are some caveats to such analysis. First, the variable depth of phenotypic information could affect the clustering, e.g., the unavailability of nerve conduction studies in BAB9484 affecting phenotypic match to previously reported YUHAL probands which were extensively evaluated clinically [36]. Another caveat is that portions of these proband phenotypes may be due to genes encompassed by CNV that are not yet associated with disease in humans or by other yet unrecognized genetic modifiers/pathogenic SNV contributing to the phenotype. Nevertheless, aggregate data of quantitative phenotypic analysis of M*dn*CNV probands suggests that at least some phenotypic variability is likely explained by dosage changes of genes encompassed by CNV genome-wide in probands. These cases also highlight the possibility of multiple genes, in one case as part of a contiguous gene duplication of *PMP22* and *RAI1* and in another as duplications of *NSD1* and *SMARCC2* at separate loci undergoing *de novo* gains in a single generation to contribute to phenotype manifestation.

## Conclusions

Characterization of the M*dn*CNV phenomenon using a multimodal genomic approach revealed insights that the M*dn*CNV mutational event likely occurs in the earliest post-zygotic stages of development—potentially in the pronuclear phase or during the first few cell divisions. Moreover, we show the utility of quantitative phenotypic analysis to identify contributory, disease-associated genes within a background of genome-wide *dn*CNVs and provide evidence for duplications at two genomic loci containing triplosensitive genes that contribute to the patient’s blended phenotype.

## Supplementary Information

**Additional file 1.** Supplementary methods, clinical description of BAB9637, BAB3097, and BAB9484, Table S1, S3, S5, S6, Figure S1-S9.

**Additional file 2: Table S2.**

**Additional file 3: Table S4.** *dn*SNVs in the proband (not include the *dn*SNV within 150bp to the breakpoint).

## Data Availability

The data generated or analyzed during this study are included in this published article. The array data has been submitted to GEO (GSE176427) [62]. The variant data of the study are available with ClinVar accession numbers: SCV002576315—SCV002576337.

## Abbreviations

aCGH: Array comparative genomic hybridization
*dn*CNVs: *De novo* copy number variants
DNMs: *De novo* Mutations
*dn*SNVs: *De novo* single-nucleotide variants
HPO: Human Phenotype Ontology
MMBIR: Microhomology-mediated break-induced replication
MPV: Multilocus pathogenic variation
NHEJ: Non-homologous end joining
OFC: Occipital frontal circumference
SNPs: Single-nucleotide polymorphisms
