# Supplementary material for: The multiple de novo copy number variant (MdnCNV) phenomenon presents with peri-zygotic DNA mutational signatures and multilocus pathogenic variation
Source: Genome Med. 2022 Oct 27;14:122. doi: 10.1186/s13073-022-01123-w (PMC9609164; doi:10.1186/s13073-022-01123-w)
Supplement: Supplementary file 1 — Additional file 1. Supplementary methods, clinical description of BAB9637, BAB3097, and BAB9484, Table S1, S3, S5, S6, Figure S1-S9. [file 13073_2022_1123_MOESM1_ESM.pdf]

Supplementary text, tables, figures, and references

The Multiple *de novo* Copy Number Variant (M*dn*CNV) phenomenon presents with peri-zygotic DNA mutational signatures and multilocus pathogenic variation

Haowei Du[1#], Angad Jolly[1,2#], Christopher M. Grochowski[1#], Bo Yuan[1,3,4], Moez Dawood[1,2,5], Shalini N. Jhangiani[5], He Li[5], Donna Muzny[5], Jawid M. Fatih[1], Zeynep Coban-Akdemir[1,6], Mary Esther Carlin[7], Angela E. Scheuerle[7], Karin Witzl[8,9], Jennifer E. Posey[1], Matthew Pendleton[10], Eoghan Harrington[10], Sissel Juul[10], P.J. Hastings[1,11], Weimin Bi[1,3], Richard A Gibbs[1,5], Fritz J Sedlazeck[1,5], James R. Lupski[1,5,12,13*], Claudia M. B. Carvalho[1,14*], Pengfei Liu[1,3*]

Illumina short-read sequencing

For family HOU3579, genome sequencing (GS) was performed on proband and parents' peripheral blood leukocyte-derived DNA at the Human Genome Sequencing Center (HGSC) at Baylor College of Medicine through the Baylor-Hopkins Center for Mendelian Genomics initiative.[1] Sequencing libraries were prepared with KAPA Hyper reagents and pooled for multiplexed sequencing. The pooled libraries were sequenced using the Illumina HiSeqX platform, which generated 150 bp paired-end reads. After demultiplexing, an average of 127 Gb sequence data was generated per personal genome library. Post-sequencing data were computationally analyzed using the HGSC HgV pipeline[2,3] which executed base calling, read mapping (BWA-mem), merging of calls, variant calling (xAtlas),[4] post-processing, and quality control (QC) metrics collection for all sequencing events. Post-sequencing QC was performed with Fluidigm SNPtrace and Error Rate In Sequencing (ERIS) software to ensure sample identity and integrity.[5,6]

For the other MdnCNV families (BAB3097, BAB3596, mCNV3/BAB9484, and mCNV7) and anonymized samples included, GS was performed using a separate protocol. The library was prepared using a PCR-free 550-bp insert size protocol by the KAPA Hyper Prep kit. The library is subjected to sequence analysis on Illumina NovaSeq 6000 platform for 150 bp paired-end reads. The following quality control metrics of the sequencing data are generally achieved: average sequenced coverage over the genome > 40X, >97.5% target base (digital exome) covered at >20X. Data analysis and interpretation are performed by the Baylor Genetics analytics pipeline. The output data from the Illumina NovaSeq are converted from BCL files to FastQ files according to each sample's specific adapter sequence using Illumina's recommended procedure. FastQ data are aligned to the human reference genome build GRCh38 using the Illumina Dragen BioIT Platform. The output of the alignment is a BAM file; QC metrics of the map-align process are recorded for

quality review. QC statistics include coverage for the digital exome regions (all coding regions, UTRs of genes, noncoding genes or intronic regions that are known to be implicated in human disease), mate-pair alignment information as well as number of total and duplicate reads. Variant calling on the BAM file is performed using the Illumina Dragen haplotype-based variant calling system and the output is a VCF file. Variant calling for copy number analyses is performed using the Illumina Dragen genome wide depth based CNV caller with custom modifications from Baylor Genetics. Structural variant calling is performed using the Illumina Manta Structural Variant Caller.

Nanopore trio long-read sequencing and mapping

LR sequencing libraries were generated using the ONT ligation sequencing kit and then sequenced with the PromethION Beta platform.[7] Sequencing depth was calculated on the resulting alignments using mosdepth v0.2.3[8] with the parameters '-F 3588 -Q 1', which calculate coverage of depth in 100 bp bins and included only primary and supplemental alignments. Alignment of LRs was performed with NGMLR v0.2.7[9] using default parameters along with the '-bam -fix' parameter for long CIGAR string support.

Clinical description of BAB9637, mCNV3/BAB9484 and BAB3097

BAB9637 is a 14-year-old male at the last clinical evaluation who was born at 38 weeks gestational age via Cesarean-section to a healthy 37-year-old mother (BAB9638) and 36-year-old father (BAB9639). The proband birth weight was 2.72 kg (Z= -1.36). Other neonatal anthropometric measurements were not reported. The neonatal period was complicated only by an inguinal hernia that was surgically repaired at 21 days of life. At 10 years of age, the proband was evaluated in the genetics clinic for a history of developmental delay (DD); the DD was a sporadic trait in the family. Notably, the parents reported that the child rolled over at 4 months of age, sat

upright at 6.5 months, walked at 14 months, and spoke his first words as a 1year-old. However, he stopped talking at the age of 3 years old and only gained back some speech thereafter. Other specific developmental milestones were not reported. He failed to advance to grade 1 from kindergarten and required special education. By fifth grade, he was able to read, write, add, and multiply. Upon examination at 10 years old, he was found to have a height of 123.5 cm (Z= -3.01), a weight of 23.2 kg (Z= -2.73), and a head circumference (occipitofrontal circumference, OFC) of 52.4 (Z= -0.62). He was noted to have dysmorphic craniofacial features, including trigonocephaly, broad forehead, small widow's peak, hypertelorism with bushy eyebrows, protruding ears, and thin lips. Skin examination revealed large hyperpigmented macules. He was also noted to have proximally placed thumbs with small hands and feet, thenar and hypothenar atrophy, winged scapulae, genital hypoplasia, and thoracolumbar scoliosis. Throughout the examination, infrequent eye contact was noted, and his parents reported a prior clinical diagnosis of borderline autism.

Family history was notable for three siblings who were reported to have neurobehavioral differences: a 16-year-old sister, a 9-year-old brother, and a 6-year-old sister. The youngest sister was also reported to have autism and intellectual disability, as well as relative macrocephaly. The clinical CMA for the same sister was non-diagnostic. A high-resolution research CMA did not detect the *dn*CNV in any family members (**Figure 1c**). The mother additionally reported four spontaneous abortions.

BAB3097 was age five at the last evaluation and exhibited global developmental delay. She was able to walk with aid starting at 4.5 years of age and had a vocabulary of 10 words. She was able to understand one- or two-part commands. She had chronic kidney failure (stage 2/3 with dysplastic kidneys) treated with calcitriol. In addition, her phenotype included strabismus, a ventricular septal defect that closed spontaneously, mild hearing loss (30 dB), constipation, and

allergies with a presentation of atopic dermatitis or systemic allergic reaction. Her behavior was sociable, enjoying spending time with other children; however, she exhibited neophobia and needed time to acclimate to novel situations.

BAB9484 was born after an uncomplicated pregnancy via elective Caesarean section (weight 3kg [50 percentile], height 50cm [25th Percentile]). He developed head support at 2.5 months, and physical examination was positive for brachycephaly, hypertelorism, redundant neck skin, wide intermammillary distance, single palmar crease, and epispadias. The Echocardiogram revealed an atrial septal defect (foramen ovale type with semi-restrictive left to right shunting) and CT scan demonstrated butterfly vertebrae with a vertebral arch anomaly and communicating hydrocephalus. A G-banded karyogram revealed 46, XY,dup(1)(p31.2).

References:

1. Posey JE, O'Donnell-Luria AH, Chong JX, et al. Insights into genetics, human biology and disease gleaned from family based genomic studies. *Genet Med*. 2019;21(4):798-812.

2. Reid JG, Carroll A, Veeraraghavan N, et al. Launching genomics into the cloud: deployment of Mercury, a next generation sequence analysis pipeline. *BMC Bioinformatics*. 2014;15:30.

3. Regier AA, Farjoun Y, Larson DE, et al. Functional equivalence of genome sequencing analysis pipelines enables harmonized variant calling across human genetics projects. *Nat Commun*. 2018;9(1):4038.

4. Farek J, Hughes D, Mansfield A, et al. xAtlas: Scalable small variant calling across heterogeneous next-generation sequencing experiments. *Cold Spring Harbor Laboratory*. Published online April 5, 2018:295071. doi:10.1101/295071

5. Jun G, Flickinger M, Hetrick KN, et al. Detecting and estimating contamination of human DNA samples in sequencing and array-based genotype data. *Am J Hum Genet*. 2012;91(5):839-848.

6. Liang-Chu MMY, Yu M, Haverty PM, et al. Human biosample authentication using the high-throughput, cost-effective SNPtrace(TM) system. *PLoS One*. 2015;10(2):e0116218.

7. Carvalho CMB, Coban-Akdemir Z, Hijazi H, et al. Interchromosomal template-switching as a novel molecular mechanism for imprinting perturbations associated with Temple syndrome. *Genome Med*. 2019;11(1):25.

8. Pedersen BS, Quinlan AR. Mosdepth: quick coverage calculation for genomes and exomes. *Bioinformatics*. 2018;34(5):867-868.

9. Sedlazeck FJ, Rescheneder P, Smolka M, et al. Accurate detection of complex structural variations using single-molecule sequencing. *Nat Methods*. 2018;15(6):461-468.

Table S1

	Sheared Library			Unsheared Library			Repeated Libraries		
Patient ID	Library ID	Yield (Gbp)	Read N50 (bp)	Library ID	Yield (Gbp)	Read N50	Library ID	Yield (Gbp)	Read N50 (bp)
BAB9637	ONLL03747	82.5	9,676	ONLL03752	22.76	10,497			
BAB9638	ONLL03748*	12.05	16,562	ONLL03753	22.21	8,805	ONLL03756	55.36	17,168
BAB9639	ONLL03749	73.98	12,060	ONLL03754*	0.07	4,988	ONLL03757	40.62	15,373

* The library was repeated due to low sequenceing yield

Table S3. Detection of *dn*CNVs from the M*dn*CNV case by array platforms and WGS

Locus	Size (kb)	Type	Array Detection		WGS Detection		Parent of Origin
			Clinical CMA	aCGH 1M	Parliament 2 SV calls	Sniffle SV calls	
4q31.22q31.23	904.4	Dup	Y	Y	Y	Y	Pat
5q35.2q35.3	927.2	Dup	Y	Y	Y	Y	Mat
6p24.2p24.1	1041.6	Dup	Y	Y	Y	Y	Pat
10q26.13q26.2	920.8	Dup	Y	Y	Y	Y	Mat
12q13.2q13.3	899.1	Dup	Y	Y	Y	Y	Pat
13q33.3q34	934.3	Dup	Y	Y	Y	Y	Mat
14q21.1	911.1	Dup	Y*	Y	Y	Y	Pat
21q21.3	1033.9	Dup	Y	Y	Y	Y	Mat

Dup, duplication; Pat, paternal; Mat; maternal. * Not reported in the clinical report due to lack of genes in the region.

Table S5. Evidence implicating *dn*SNV as a mutational signature of SV mutagenesis

Phasing Information	Size of Window (Mb)	DNM present near bkp	Size of region at bkp (Mb)	Mappable region (Mb)	DNM density near bkp	Baseline DNM density	p-value compared to baseline	Local DNM density in population	p-value compared to population
Unphased	1	4	47.1	5826.0	8.48e-08	1.56e-8	0.001	2.86e-08	0.01
	4	7	143.1	5826.0	4.89e-8	1.56e-8	0.0022	1.50e-08	0.002
Paternal	1	2	11.8	2913.0	5.59e-8	2.52e-8	0.0032	-	-
	4	4	35.8	2913.0	1.12e-7	2.52e-8	0.0021	-	-
Maternal	1	1	11.8	2913.0	8.46e-8	0.67e-08	0.0029	-	-
	4	1	35.8	2913.0	2.33e-08	0.67e-08	0.024	-	-

DNM, *de novo* mutation; bkp, breakpoint.

Table S6. Similarity score of *dn*CNV encompassing genes

**BAB9637**				
**Gene name**	**Sim.Score[a]**	**pLI**	**Gene combinations[b]**	**Sim.Score**
NSD1	0.60440297	1.00	*SMARCC2 NSD1*	0.64233316
SMARCC2	0.59028067	1.00	*EDNRA NSD1*	0.61928193
RPS26	0.49492267	0.75	*RPS26 NSD1*	0.61172692
EDN1	0.42369687	0.08	*NSD1 UROS*	0.60755226
UROS	0.41187302	0.01	*ERBB3 NSD1*	0.60578791
COL4A1	0.40487663	1.00	*SNCB NSD1*	0.60519053
SUOX	0.3800252	0.00	*SLC39A5 NSD1*	0.60440297
EDNRA	0.37505858	0.99	*MIP NSD1*	0.60326677
COL4A2	0.35768528	0.00	*EDN1 NSD1*	0.60302861
SNCB	0.29585011	0.20	*COL4A1 NSD1*	0.60295504
**BAB3097**				
**Gene name**	**Sim.Score**	**pLI**	**Gene combinations**	**Sim.Score**
RAI1	0.51732851	1.00	*RAI1 SREBF1*	0.58188604
FLCN	0.49762947	0.96	*FLCN SREBF1*	0.57733223
SREBF1	0.47257009	0.99	*SPEN SREBF1*	0.57650011
SPEN	0.45450513	1.00	*GABRB2 SREBF1*	0.56529538
GABRB2	0.4414777	0.94	*RAI1 MYOCD*	0.56370926
CLCN6	0.41304961	0.16	*GABRA1 SREBF1*	0.55058737
PMP22	0.38805043	0.83	*MFN2 SREBF1*	0.54878793
GABRG2	0.38795987	0.96	*RAI1 GABRG2*	0.54609193
TNFRSF13B	0.38458958	0.00	*PMP22 SREBF1*	0.54557315
MYOCD	0.37842175	0.93	*GABRB2 MYOCD*	0.54331673
			GABRG2 SREBF1	0.5425714
			FLCN TNFRSF13B	0.54148817
			CLCN6 RAI1	0.54123533
			RAI1 FLCN	0.53994127
**mCNV3/BAB9454**				
**Gene name**	**Sim.Score**	**pLI**	**Gene combination**	**Sim.Score**
TBL1XR1	0.50162636	1.00	*GATA5 TBL1XR1*	0.56344932
EXT2	0.3998587	0.00	*NEXN TBL1XR1*	0.53131955
TBCE	0.38962846	0.00	*EXT2 TBL1XR1*	0.5286138
ALX4	0.38780128	0.23		
GATA5	0.32905259	0.25		
SLC35D1	0.28990795	0.00		
EEF1A2	0.27958557	0.96		
B3GALNT2	0.26285315	0.00		
NEXN	0.25184031	0.00		
ASPRV1	0.24495963	0.09		
a Rank ordered lin similarity score; b Gene combinations with more than 5% increasement of similarity score were listed.				

Chr4_junction_hg38 blunt junction

Direct repeat → Direct repeat →

```
Chr4: 148399131-148399205 (+)
TTGCTTTGCATCCCTTAAAACAGTGGCCTCTTGCCTCTGGCCTTTGTGTTAACTGTCTCAGTAGGCCTGCTTGTAAACTGTCTCAGTAGGCCTGCTTGTA
TTGCTTTGCATCCCTTAAAACAGTGGCCTCTTGCCTCTGGCCTTTGTGTTTCCTGGGTAAACAAGGAAAACCTATATCCCAACACCTAGGGAGGCAGAGG
CATAAACCAGGACTGTATTATGTCAGGACAATAGGCATAAACCAGGACTGTCCTGGGTAAACAAGGAAAACCTATATCCCAACACCTAGGGAGGCAGAGG
chr4: 147494705-147494925 (+)
```

Inverted repeat

Chr5_junction_hg38 microhomeology

```
Chr5:177376796-177376856(+)
CATCTTAGCTTTAATTTTTTTAAGGATACAATTTTTAATCATTACAATAATGCGAAGGACTCCCCAAGATCCCTCTTTGTCCCTGATGGTCCACCATTCTTTGG
CATCTTAGCTTTAATTTTTTTAAGGATACAATTTTTAATCATTACAATAATGCGTCTCAAAAAAAAAAAAATCTACCTCCTGAGGATGTTCGGGAGATCAAATG
TGAGCCGAGATCGTGCCACTGCACTCCAGCCTGGGCAACAAGAGTGAAACTcCGTCTCAAAAAAAAAAAAATCTACCTCCTGAGGATGTTCGGGAGATCAAATG
chr5: 176449518-176449621(+)
```

Chr6_junction_hg38 1bp insertion

```
Chr6:12522836-12522935(+)
TTTAGTCTAATTAGGTCCCATTTGTCAATTTGTCTTTTTATTGCTATTGCTTCTGCCATCTTCATCATGAAATCTTTGCCAGGACCTATGTCCTGAATGG
TTTAGTCTAATTAGGTCCCATTTGTCAATTTGTCTTTTTATTGCTATTGCAATAAAACAGCAAAGAATGGTGCCAGAGTTTAATTAATAGGATAAaGTTA
GTACCTAGGAACTCCAAAGAACAAACTGTTGCTCGTGCAAAATTAAATTTCATAAAACAGCAAAGAATGGTGCCAGAGTTTAATTAATAGGATAAGGTTA
Chr6:11481258-11481357(+)
```

Chr10_junction_hg38 microhomology/microhomeology

```
Chr10:125841639-125841747(+)
TCTCTGCTCTGTGCTCCTCAAAATATAATTTCAAATGGATCCGATCTAAATCTATACCTAGTGCAGATTTGCAAAAAAAGAAAAGGAATAGTCATTAAGGATACCTGTT
TCTCTGCTCTGTGCTCCTCAAAATATAATTTCAAATGGATCCGATCTAAATCTATACCT---------------------ATATATTCAGCACTGAAAATATGAATACA
TATTTTGCTTCCCAACTAGTTTTTAAGACTTCTGCCAAATGGGTATATCTCTATACC-ctgtatctatctatggagaccATATATTCAGCACTGAAAATATGAATACA
Chr10:124920797-124920904(+)
```

Inverted repeat

Chr12_junction_hg38 microhomology (100%)

```
Chr12:56885539-56885640(+)
AGGAGTAGGGCATTGCTATAAATATACCTGAAGTAGGGCATTGTTATAAATATACCCGAAAATGTGAAAGAAACTTTGGAACTAGGTAATGAGCAGAGGTTG
AGGAGTAGGGCATTGCTATAAATATACCTGAAGTAGGGCATTGTTATAAATACCGGTGGGTGGATCTGGATATTCTTCAGGCCAGCTAAAGGTTGATCACTT
AAAAGAAGAGCACAGCACCAGGATGCCAAGGAGATACTGGGGATCTAGGGTACCGGTGGGTGGATCTGGATATTCTTCAGGCCAGCTAAAGGTTGATCACTT
Chr12:55986462-55986563(+)
```

Chr13_junction_hg38 1bp microhomology

```
Chr13:110341087-110341186(+)
AATCACGACAGAGAAGGGAAAGAAGCCCGCCCGCCACTTCCGGTGTTCACTGAGCAGGTCGGTGGCCAGCGCTGCCTTCGCATCAGAACCCCATTTTAAA
AATCACGACAGAGAAGGGAAAGAAGCCCGCCCGCCACTTCCGGTGTTCACATGGCACATGGTACAGGGCATGAGTGTTTCCTTGAACCACACTGGAAATC
AGCTGGGGCAGCTCAGCATTGCACGAGGCAAGCTGGGTGCAAAGAGGCTCATGGCACATGGTACAGGGCATGAGTGTTTCCTTGAACCACACTGGAAATC
chr13:109406785-109406884(+)
```

Chr14_junction_hg38 microhomology (100%)

```
Chr14:41479652-41479753 (+)
GACAGAGATACTCTCATGCTGTTTCACAGGCCAGGCATGCTTATTCTCCAGTATTAGGTTCAGGTTGTCTAACTCAGGGTTCCTCCCTTTTATGCAGTCCCA
GACAGAGATACTCTCATGCTGTTTCACAGGCCAGGCATGCTTATTCTCCAGTATAACCATTCTTCAGTATAAAGTAGATAACAAAATACATTCCACTCAGTT
CATGAGACCAAGCCATTAGCCATCACATATTAATTTGTGCAAATCCATATGTATAACCATTCTTCAGTATAAAGTAGATAACAAAATACATTCCACTCAGTT
chr14:40568543-40568644 (+)
```

Chr21_junction_hg38 microhomology (100%)

```
Chr21:29192247-29192350 (+)
GACTTGTCTCGAACTCCCAACCTCAGGTGATCCGCCCACCTCGGCCTCTCAAAGTGCTGGGATTATAGGCGTGAGCCACCGCACCCGGCCAACAAACACAAATT
GACTTGTCTCGAACTCCCAACCTCAGGTGATCCGCCCACCTCGGCCTCTCAAAGAAGGCTTCTGTGACCAAATGTGAGCGGAGGGAGCTTTTCCCCCCACCAAG
AACTCTCAAACTATGTTTTTCATCTCCTCTCACCCACAGCAAACATCAAAAAAGAAGGCTTCTGTGACCAAATGTGAGCGGAGGGAGCTTTTCCCCCCACCAAG
Chr21:28158293-28158396 (+)
```

Figure S1

Figure S1. Breakpoint junction sequence aligned with the distal and proximal reference sequence. Base pair level resolution of breakpoint junction with non-B DNA formation motif highlighted for all eight duplications.

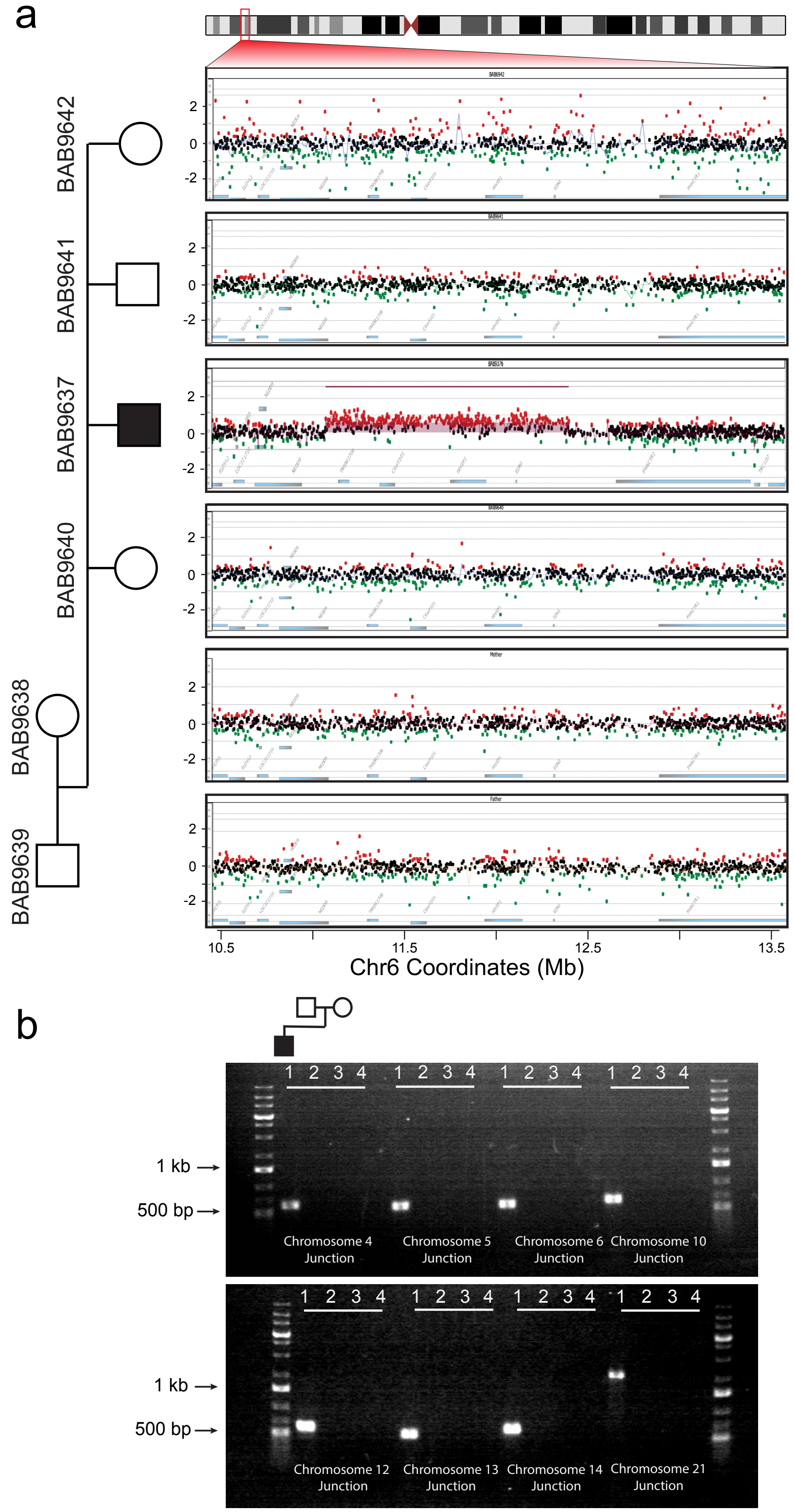
a
BAB9642
BAB9641
BAB9637
BAB9640
BAB9638
BAB9639
2
0
-2
10.5
11.5
12.5
13.5
Chr6 Coordinates (Mb)
b
1 2 3 4
1 kb
500 bp
Chromosome 4 Junction
Chromosome 5 Junction
Chromosome 6 Junction
Chromosome 10 Junction
Chromosome 12 Junction
Chromosome 13 Junction
Chromosome 14 Junction
Chromosome 21 Junction

Figure S2

Figure S2. SV mutagenesis in family HOU3579 with a) representative *de novo* duplication and b) M*dn*CNV breakpoint junctions observed only in the proband genome. a From top to bottom shows observed copy number state of region 6p24.2p24.1 (see all *dn*CNV in the Additional file: Figure S4) from 1 M array CGH data with the pedigree aligned to the left indicating corresponding individuals. **b** Observed PCR products of all breakpoint junctions in the order of proband (lane 1), father (lane 2), mother (lane 3), and control (lane 4). Expected PCR product size for each breakpoint are as follows: 587bp (Chr4), 586 bp (Chr5), 530 bp (Chr6), 598 bp (Chr10), 543 bp (Chr12), 502 bp (Chr13), 596 bp (Chr14), 1300 bp (Chr21). A 1 kb ladder was run in the first and last lanes of each gel.

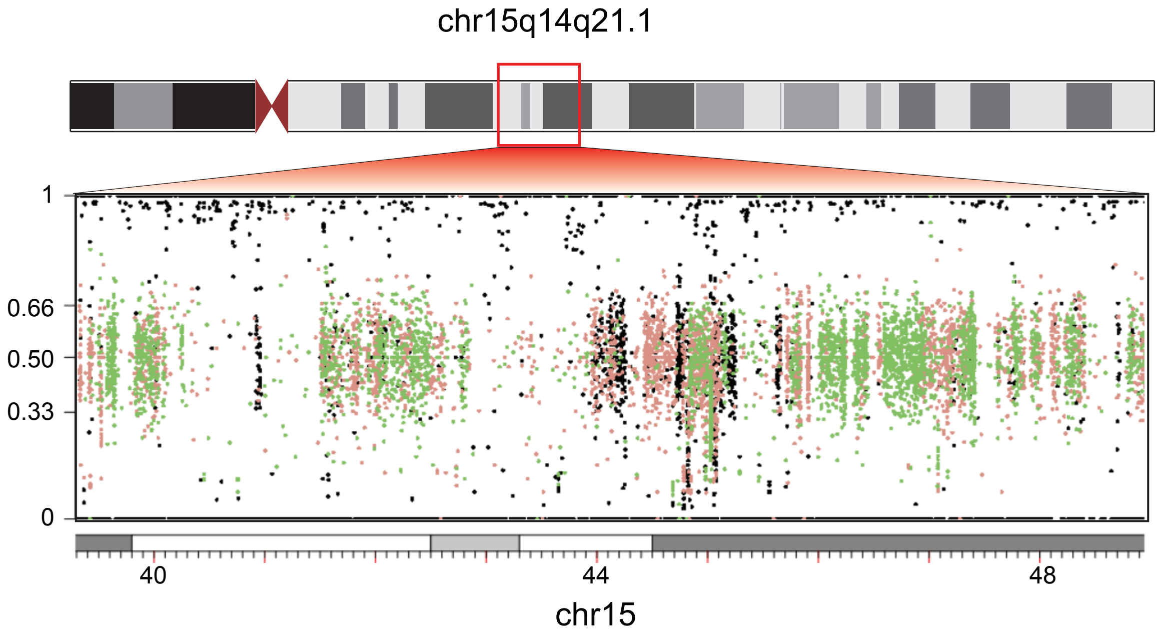

Figure S3

Figure S3. WGS B-allele frequency did not detect the ~7 Mb region of AOH on 15p14p21.1. Horizontally oriented chromosome 15 ideogram (top) with genomic interval (box in red) of 15p14p21.1 expanded. Observed B-allele frequency with phased information (bottom); green dots represent paternally inherited B-allele frequency for individual informative SNPs, while pink dots represent maternally inherited B-allele frequency.

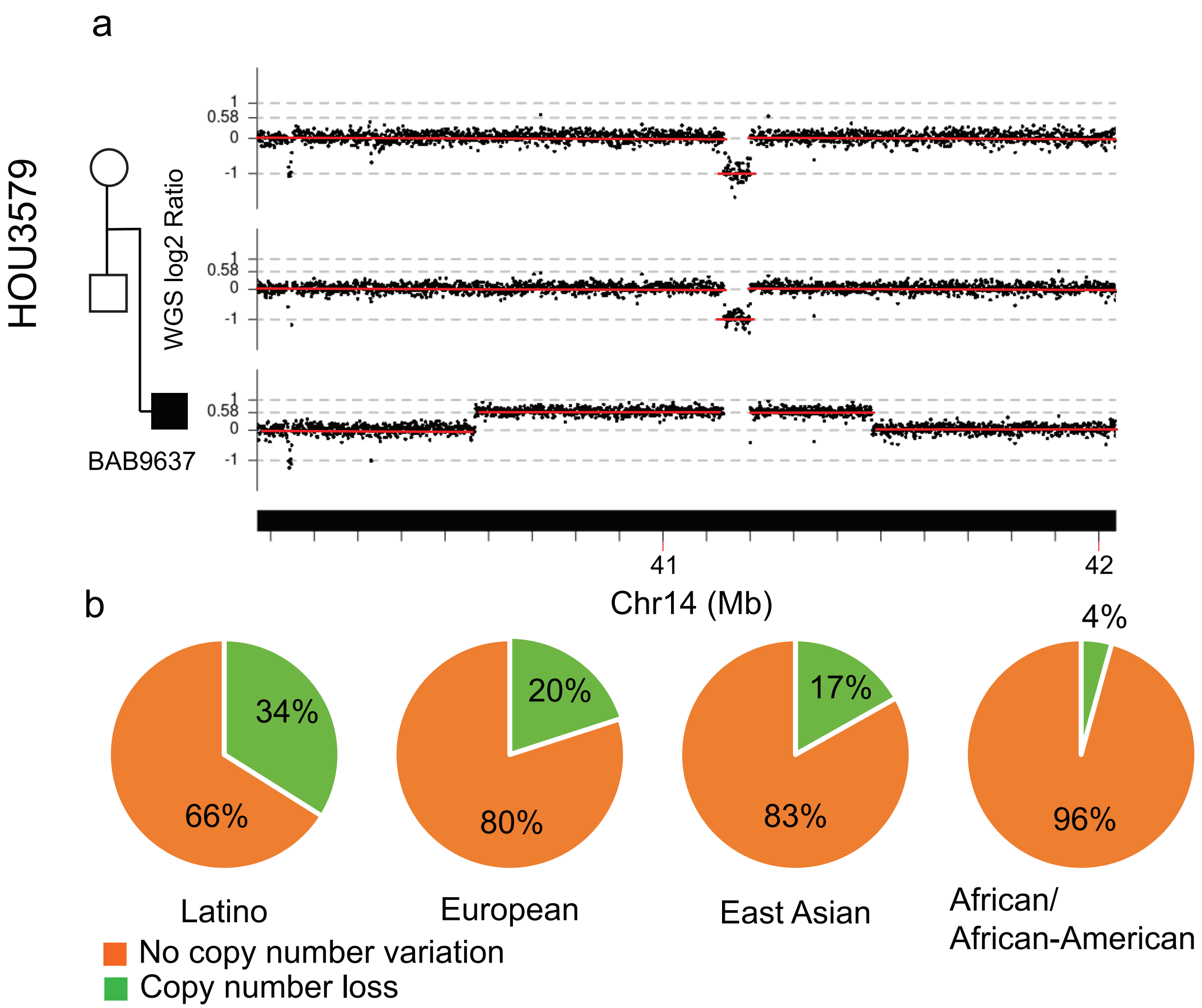
a
HOU3579
BAB9637
WGS log2 Ratio
1
0.58
0
-1
41
42
Chr14 (Mb)
b
34%
66%
Latino
20%
80%
European
17%
83%
East Asian
4%
96%
African/
African-American
No copy number variation
Copy number loss

Figure S4

Figure S4. An observed 60.2 kb homozygous deletion within the *de novo* duplication. Log2 ratio of read depth from SR 40x genome sequencing (GS); red horizontal line reveals the average depth of coverage consistent with copy number changes. **b** Frequency of CNV in gnomAD SV database.

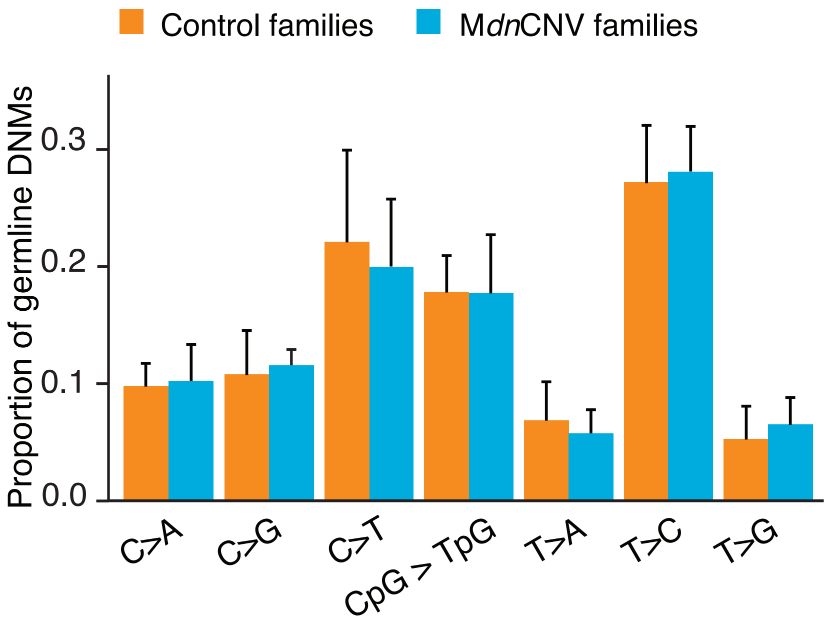

Figure S5

Figure S5. *De novo* substitution mutational spectra of MdnCNV families (Blue) and anonymized control families (Orange).

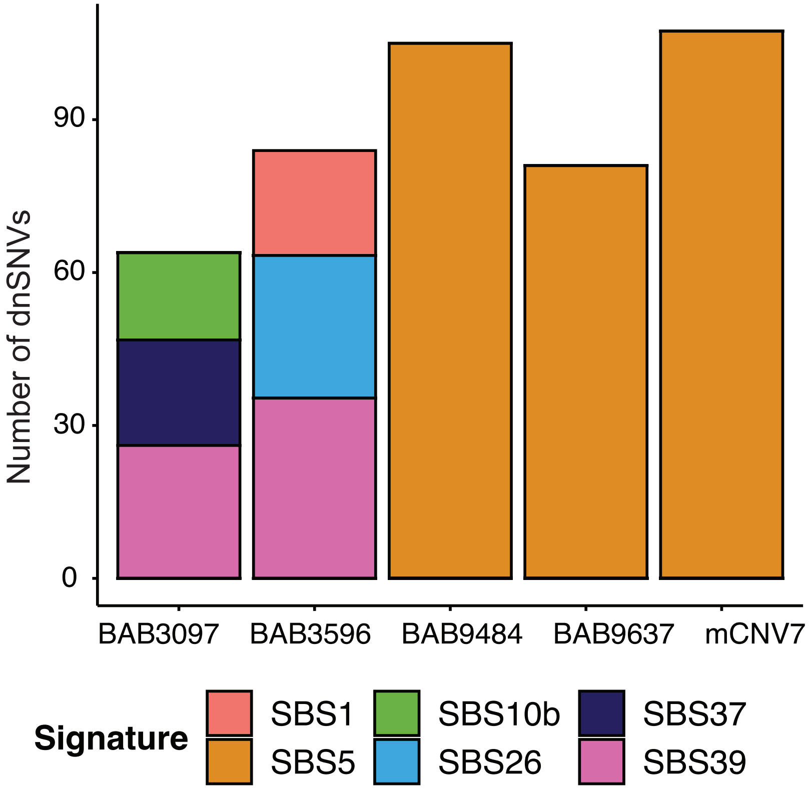

Figure S6

Figure S6. Bar plot shows the contribution of SBS signatures refitted by *dn*SNV outside of *dn*CNV region.

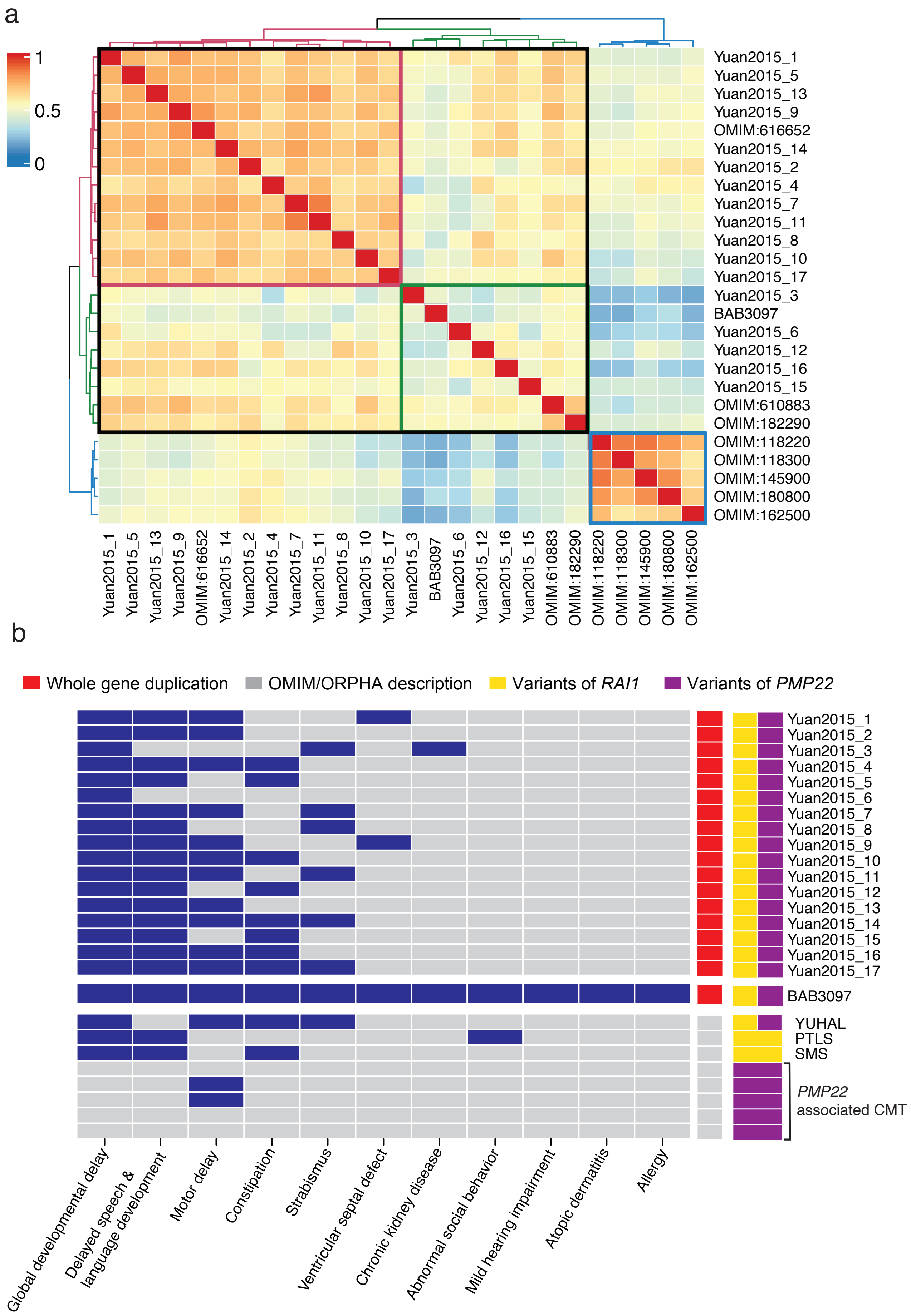
a
1
0.5
0
Yuan2015_1
Yuan2015_5
Yuan2015_13
Yuan2015_9
OMIM:616652
Yuan2015_14
Yuan2015_2
Yuan2015_4
Yuan2015_7
Yuan2015_11
Yuan2015_8
Yuan2015_10
Yuan2015_17
Yuan2015_3
BAB3097
Yuan2015_6
Yuan2015_12
Yuan2015_16
Yuan2015_15
OMIM:610883
OMIM:182290
OMIM:118220
OMIM:118300
OMIM:145900
OMIM:180800
OMIM:162500
b
Whole gene duplication
OMIM/ORPHA description
Variants of RAI1
Variants of PMP22
Yuan2015_1
Yuan2015_2
Yuan2015_3
Yuan2015_4
Yuan2015_5
Yuan2015_6
Yuan2015_7
Yuan2015_8
Yuan2015_9
Yuan2015_10
Yuan2015_11
Yuan2015_12
Yuan2015_13
Yuan2015_14
Yuan2015_15
Yuan2015_16
Yuan2015_17
BAB3097
YUHAL
PTLS
SMS
PMP22 associated CMT
Global developmental delay
Delayed speech & language development
Motor delay
Constipation
Strabismus
Ventricular septal defect
Chronic kidney disease
Abnormal social behavior
Mild hearing impairment
Atopic dermatitis
Allergy

Figure S7: Phenotype similarity score analysis for disease associated genes and potential gene combination for BAB3097. a Heatmap representing color-coded Lin semantic similarity scores of BAB**3097**. Both rows and columns are clustered using ward method. Dendrogram is present at top and to the left of the heatmap. **b** Annotation grid demonstrates (from top to bottom) reported YUHAL proband, BAB3097, OMIM *RAI1/PMP22* associated phenotypes, blue squares indicate the presence of the phenotype, i.e., HPO term, while grey represents the absence of the term. Two colored columns are present at right of the grid, the first colored column denotes variant type and the second denotes whether an individual has a variant affecting *RAI1* (purple) or *PMP22* (yellow). The grey colored bars in the first colored column denote OMIM annotated *RAI1* and *PMP22* phenotypes.

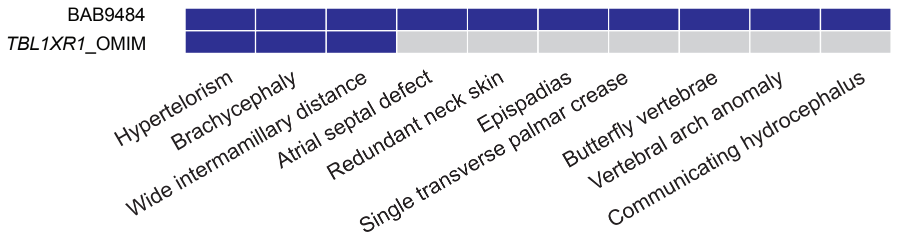
BAB9484
TBL1XR1_OMIM
Hypertelorism
Brachycephaly
Wide intermamillary distance
Atrial septal defect
Redundant neck skin
Epispadias
Single transverse palmar crease
Butterfly vertebrae
Vertebral arch anomaly
Communicating hydrocephalus

Figure S8: Phenotype similarity score analysis for disease associated genes and potential gene combination for BAB3097. a Heatmap representing color-coded Lin semantic similarity scores of BAB**3097**. Both rows and columns are clustered using ward method. Dendrogram is present at top and to the left of the heatmap. **b** Annotation grid demonstrates (from top to bottom) reported YUHAL proband, BAB3097, OMIM *RAI1/PMP22* associated phenotypes, blue squares indicate the presence of the phenotype, i.e., HPO term, while grey represents the absence of the term. Two colored columns are present at right of the grid, the first colored column denotes variant type and the second denotes whether an individual has a variant affecting *RAI1* (purple) or *PMP22* (yellow). The grey colored bars in the first colored column denote OMIM annotated *RAI1* and *PMP22* phenotypes.

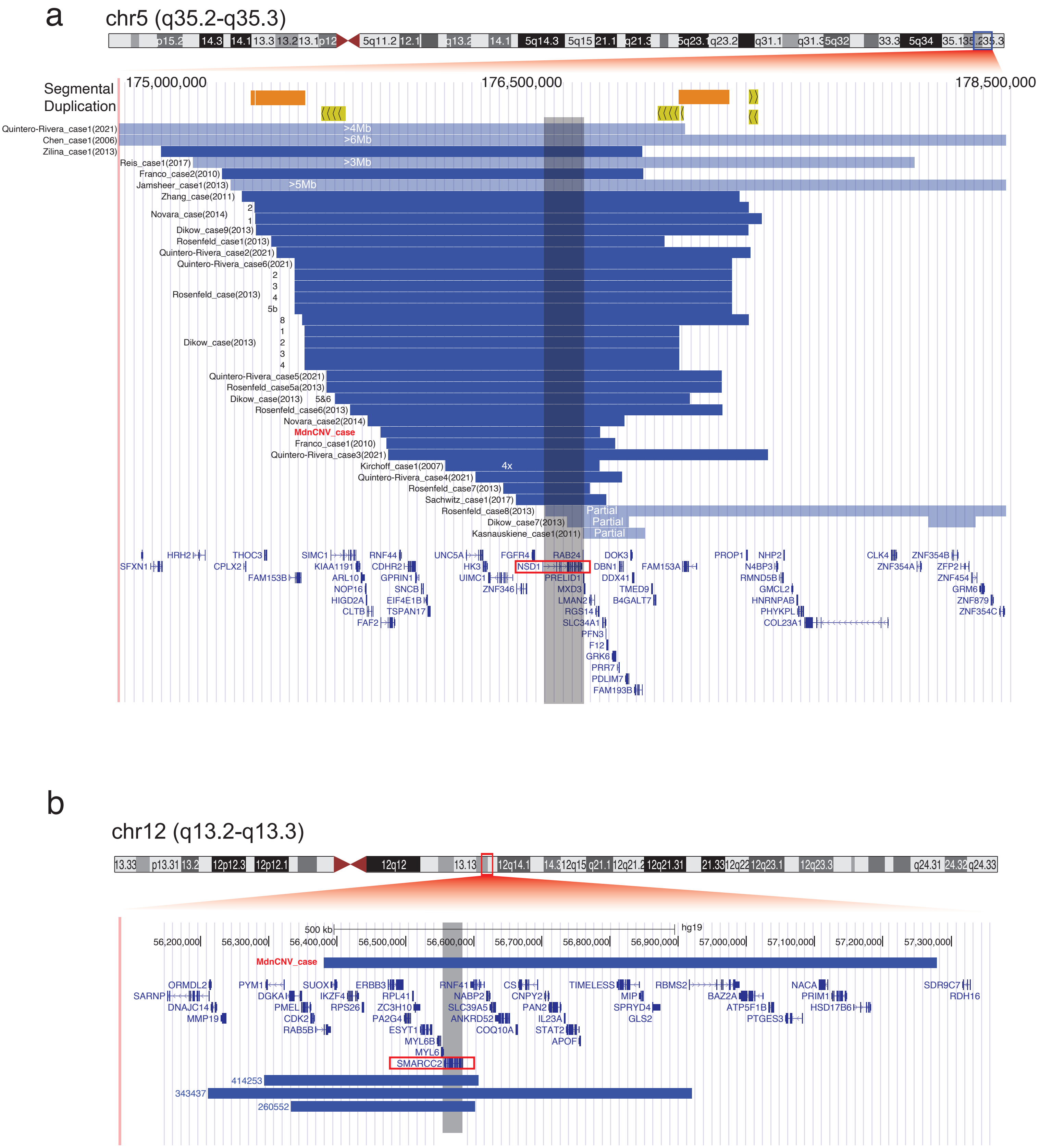
a
chr5 (q35.2-q35.3)
Segmental Duplication
175,000,000
176,500,000
178,500,000
Quintero-Rivera_case1(2021)
Chen_case1(2006)
Zilina_case1(2013)
Reis_case1(2017)
Franco_case2(2010)
Jamsheer_case1(2013)
Zhang_case(2011)
Novara_case(2014)
Dikow_case9(2013)
Rosenfeld_case1(2013)
Quintero-Rivera_case2(2021)
Quintero-Rivera_case6(2021)
Rosenfeld_case(2013)
Dikow_case(2013)
Quintero-Rivera_case5(2021)
Rosenfeld_case5a(2013)
Rosenfeld_case6(2013)
Novara_case2(2014)
MdnCNV_case
Franco_case1(2010)
Quintero-Rivera_case3(2021)
Kirchoff_case1(2007)
Quintero-Rivera_case4(2021)
Rosenfeld_case7(2013)
Sachwitz_case1(2017)
Rosenfeld_case8(2013)
Dikow_case7(2013)
Kasnauskiene_case1(2011)
>4Mb
>6Mb
>3Mb
>5Mb
4x
Partial
NSD1
b
chr12 (q13.2-q13.3)
500 kb
hg19
56,200,000
56,300,000
56,400,000
56,500,000
56,600,000
56,700,000
56,800,000
56,900,000
57,000,000
57,100,000
57,200,000
57,300,000
MdnCNV_case
SMARCC2
414253
343437
260552

Figure S9

Figure S9. The smallest region of overlapping duplication at 5q35.2q35.3 and 13q33.3q34. Horizontally oriented chromosome ideogram (top) with genomic interval (boxed in red). **a.** Below ideogram reported duplications (blue) are aligned. Segmental duplications are colored in organ and yellow. Encompassed genes are at the very bottom. **b.** The same structure as (**a**) is used to depict SMARCC2 duplication reported in Decipher database.
